# An Equivalent Substitute Strategy for Constructing 3D Ordered Porous Carbon Foams and Their Electromagnetic Attenuation Mechanism

**DOI:** 10.1007/s40820-022-00900-x

**Published:** 2022-08-02

**Authors:** Meng Zhang, Hailong Ling, Ting Wang, Yingjing Jiang, Guanying Song, Wen Zhao, Laibin Zhao, Tingting Cheng, Yuxin Xie, Yuying Guo, Wenxin Zhao, Liying Yuan, Alan Meng, Zhenjiang Li

**Affiliations:** 1 College of Materials Science and Engineering, College of Electromechanical Engineering, Qingdao University of Science and Technology, Qingdao, 266061 People’s Republic of China; 2 State Key Laboratory Base of Eco-Chemical Engineering, College of Chemistry and Molecular Engineering, College of Chemical Engineering in Gaomi Campus, Qingdao University of Science and Technology, Qingdao, 266042 People’s Republic of China

**Keywords:** Porous carbon foam, Electromagnetic wave absorption, Adjustable pore structure, Polarization loss, Attenuation mechanism

## Abstract

Three-dimensional (3D) ordered porous carbon is generally believed to be a promising electromagnetic wave (EMW) absorbing material. However, most research works targeted performance improvement of 3D ordered porous carbon, and the specific attenuation mechanism is still ambiguous. Therefore, in this work, a novel ultra-light egg-derived porous carbon foam (EDCF) structure has been successfully constructed by a simple carbonization combined with the silica microsphere template-etching process. Based on an equivalent substitute strategy, the influence of pore volume and specific surface area on the electromagnetic parameters and EMW absorption properties of the EDCF products was confirmed respectively by adjusting the addition content and diameter of silica microspheres. As a primary attenuation mode, the dielectric loss originates from the comprehensive effect of conduction loss and polarization loss in S-band and C band, and the value is dominated by polarization loss in X band and Ku band, which is obviously greater than that of conduction loss. Furthermore, in all samples, the largest effective absorption bandwidth of EDCF-3 is 7.12 GHz under the thickness of 2.13 mm with the filling content of approximately 5 wt%, covering the whole Ku band. Meanwhile, the EDCF-7 sample with optimized pore volume and specific surface area achieves minimum reflection loss (RL_min_) of − 58.08 dB at 16.86 GHz while the thickness is 1.27 mm. The outstanding research results not only provide a novel insight into enhancement of EMW absorption properties but also clarify the dominant dissipation mechanism for the porous carbon-based absorber from the perspective of objective experiments.

**Supplementary Information:**

The online version contains supplementary material available at 10.1007/s40820-022-00900-x.

## Highlights

- Three-dimensional ordered porous carbon foam with abundant hetero-atoms is successfully prepared through employing silica microspheres template.
- The pore volume and specific surface area are effectively adjusted by an equivalent substitute strategy to investigate their influence on electromagnetic wave absorption performances.
- The attenuation mechanism of electromagnetic wave is clarified according to the contribution of conduction loss and polarization loss on dielectric loss.

**Supplementary Information:**

The online version contains supplementary material available at 10.1007/s40820-022-00900-x.

## Graphical abstract

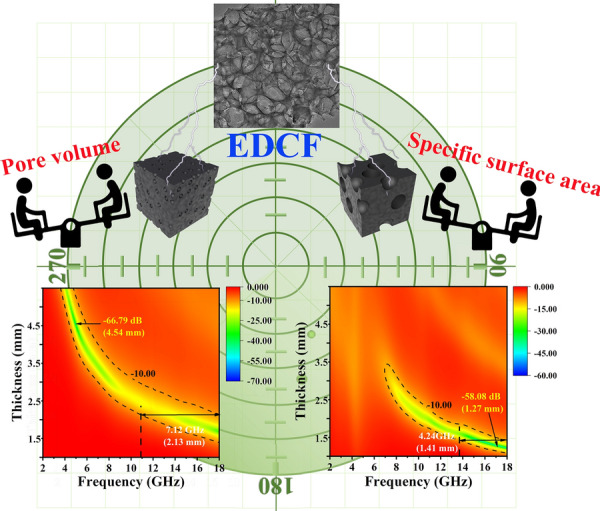

## Introduction

Currently, with the explosive growth of the semiconductor industry and wireless communication technology, electromagnetic radiation and electromagnetic interference are becoming increasingly rampant, which not only affects the normal operation of precision electronic instruments but also poses a serious threat to the health of biological systems [1–3]. In this case, it is extremely urgent to solve the environmental problems of electromagnetic pollution, building an effective protective barrier for people, and ensure the security of private information [4, 5]. Fortunately, electromagnetic wave (EMW) absorbing material can convert electromagnetic energy into other forms, being promoted to a prominence position by countries around the world and becoming a priority development field [6–8].

Experiencing the prosperity of metals and their oxides in the early stage, the industrial community proposes new requirements of "thin, light, wide and strong" for EMW absorbing materials, that is, thin coating thickness, lightweight, wide absorption bandwidth and strong absorption capacity [9–11]. Deservedly, researchers place great expectations on the development of dielectric loss EMW absorbing materials, especially carbonaceous materials with low density, strong corrosion resistance, high thermal stability, good electrical conductivity and adjustable dielectric properties [12–14]. Meanwhile, carbon materials also have the characteristics of diverse microstructure, convenient utilize, simple maintenance, good compatibility with the matrix material etc., which is incomparable to many materials. Over the past decade, a variety of carbonaceous materials, such as carbon nanotubes, carbon fiber, graphene, and carbon quantum dots, have been developed to fight against electromagnetic pollution [15–19].

While retaining the universal advantages of carbon family, three-dimensional (3D) ordered porous carbon nanomaterial with abundant pore structure not only further lowers the density, but also improves the impedance matching of EMW absorbing material, and acquires better multiple reflection and scattering for incident EMWs, being regarded as one of the ideal candidates for the novel EMW absorbing material [6, 20, 21]. As a result, various strategies (including nanowire/nanosheet self-assembly, sacrifice templates, and biomass derivation/freeze drying) have been designed and implemented to construct the 3D ordered porous carbon-based EMW absorbing materials, and some promising reflection loss (RL) and effective absorption bandwidth (EAB) measurements have been reported, which is also constantly updated [22]. Nitrogen-doped 3D porous reduced graphene oxide aerogels were prepared by Shu and colleagues. By adjusting the doping amount of nitrogen atoms, the microwave absorption performances can be controlled effectively. The measured minimal reflection loss of − 56.4 dB and effective absorption broad of 6.8 GHz were achieved at the doping amount of 9.41% [23]. Besides, employing abandoned polyurethane as the sacrificial template of a three-dimensional carbon network, Zhu et al. prepared 3D Fe/Fe_2_O_3_@porous carbon nanocomposites by simple hydrothermal and thermal treatments, and the optimal sample has a minimum RL of − 54.7 dB at a thickness of 1.4 mm and an EAB of 6.4 GHz at 1.9 mm [24]. With wheat straw as raw material, Aslam et al. produced a 3D carbon foam that obtained a minimal reflection loss of − 37 dB at 12.1 GHz with the matching thickness of 2.0 mm. At the same time, a wide EAB of 8.8 GHz has also been measured at a matching thickness of 2.5 mm [25]. While Zhao et al. employed peach gum as raw material to prepare honey-comb carbon, which was featured with a 3D porous structure and a reflection loss of − 59.4 dB with the effective absorption bandwidth of 4.1 GHz at a matching thickness of 2.0 mm [26]. Nevertheless, there are still some challenges concerning 3D-ordered porous carbonaceous absorbing materials to be addressed. First of all, it is widely accepted that increasing pore volume and specific surface area is the effective approach to optimizing EMW absorption performances of 3D ordered porous carbon materials. Up to date, majority of the reported works adjust the EMW absorption properties only by simply changing the volume of the constructed pore or addition amount of template microspheres [20, 27]. The variation of pore volume also causes the fluctuation of specific surface area, which seriously interferes in the investigation concerning the influence of the latter on the EMW absorption properties of the 3D ordered porous carbon materials. Due to this, the role of the specific surface area in the EMW absorption properties is still unknown, although it is extremely important for absorbers. Secondly, people obviously invest much effort in pursuing better EMW absorption performances, which is contrast to the less attention paid to the study on the EMW attenuation mechanism of 3D ordered porous carbon materials. Their excellent EMW absorption properties are perfunctorily attributed to the synergistic effect of conduction loss and polarization relaxation caused by the porous structure, which lacks differentiation and accuracy [15, 28]. In other words, the effects of conduction loss and polarization relaxation on the enhancement of EMW absorption performance can not be expounded and enhanced in a targeted way. Therefore, the optimization of EMW absorption performance of 3D ordered porous carbon-absorbing materials can only rely on the semi-empirical attenuation mechanism [29, 30].

In order to solve the above two significant problems and sweep away the obstacle to the development of 3D ordered porous carbon-absorbing materials, the products with the controlled pore volume and specific surface area should be first prepared. It is well known that biomass-derived porous carbon with the characters, such as environment friendliness, wide sources of raw materials, feasible technique methods, high yields, low production cost and intrinsic porous structure plays an important role in EMW absorbing areas. Eggs are rich in protein, cholesterol and lecithin matter, and there are also abundant N, O, S, P, etc. hetero-atoms embedded in the derived carbon lattice, endowing the product in situ doping effect and some peculiar properties. More importantly, the egg liquid possesses good fluidity and plasticity, and the morphology and microstructure can be easily adjusted through the curing and subsequent carbonization process. In this case, the size and ratio of created pores can regulate in an effective manner, which is also beneficial for the enhancement of the interface polarization and dielectric loss, as well as EMW absorption properties. Herein, using the egg as biomass raw material and silica microspheres with the diameter of 200 nm as removable templates, a novel egg-derived porous carbon foam (EDCF) with uniform pore diameter and different pore volume was firstly prepared by adjusting the addition amount of silica. Thus, the widest effective absorption bandwidth (EAB) value of 7.12 GHz was achieved at the thickness of 2.13 mm. Following the equivalent replacement strategy, a group of EDCF samples with the same porosity (pore volume) but different specific surface area and the same specific surface area but different pore volume were also respectively constructed to investigate the influence of individual pore volume and specific surface area on the EMW absorption performances. While revealing the influence of porosity and specific surface area on electromagnetic wave attenuation, the EDCF sample with optimal porosity and specific surface area was obtained. Moreover, its minimum RL is -58.08 dB at the thickness of 1.27 mm. This work not only fills in the blank of the influence of pore volume and specific surface area on the EMW absorption performance of porous carbon nanomaterials but also provides a promising strategy for the development of ultra-light porous carbon EMW absorbing material.

## Experimental Section

**Fig. 1:**
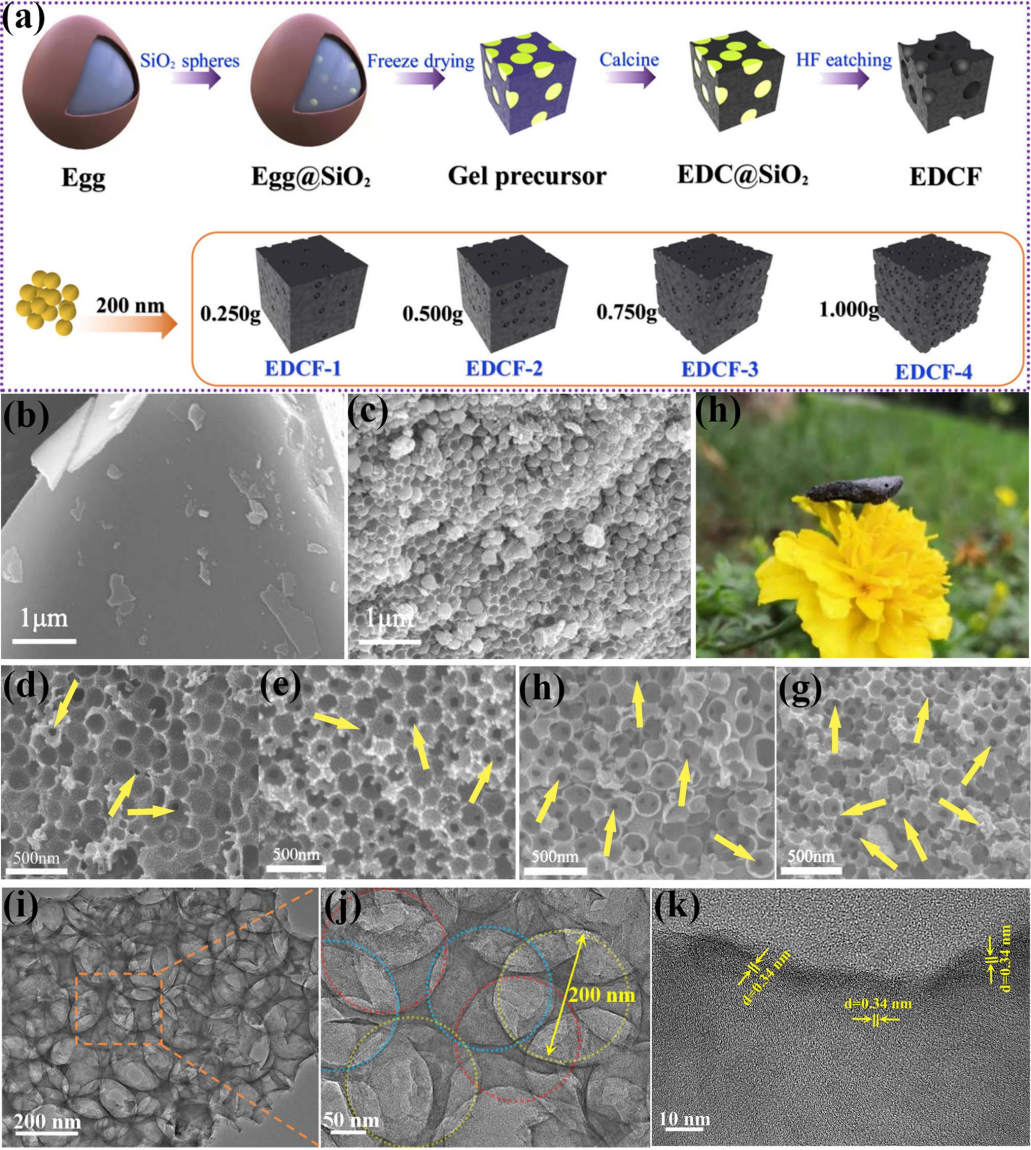
**a** Schematic illustration of the synthesis process of EDCF samples with different amounts of 200 nm silica microsphere addition; **b** SEM images of EDC and **c** EDC@SiO_2_ nanocomposites; **d**–**g** SEM images of EDCF-1 ~ EDCF-4 samples; **h** the photograph of free-standing EDCF; **i**, **j** representative TEM images and **k** HRTEM images of EDCF samples

Employing the egg as biomass raw material and the self-made silica microsphere as the template, 3D-ordered porous EDCF was prepared through freeze drying, subsequent calcination, and hydrofluoric acid etching processes. The specific steps are as follows (shown in Fig. 1a): Firstly, the uniform silica microsphere (about 200 nm in diameter) prepared by the Stöber method was introduced into a certain mass of egg liquid (about 50 g) and mechanically stirred for two hours to fully mix them [19, 31, 32]. Then, the egg liquid mixture was steamed at 100 °C for 20 min, and the cooked sample was treated for 48 h by vacuum freezing and drying technology. Subsequently, the lyophilized precursor was calcined at 850 °C for 2 h under the protection of argon atmosphere, followed by the 3D-ordered porous egg-derived carbon with embedded uniform silica microspheres (EDC@SiO_2_ nanocomposites) being collected. After soaking in hydrofluoric acid for 48 h, the silica template was removed completely, and the residual product was rinsed with deionized water several times until the solution reached neutral. Finally, the sample was dried completely, and the 3D ordered porous EDCF was obtained. In addition, a series of EDCF samples with different porosity (pore volume) were prepared by adjusting the addition amount of SiO_2_ microspheres to 0.25, 0.5, 0.75, and 1.0 g, which was named EDCF-1, EDCF-2, EDCF-3, and EDCF-4, respectively.

## Results and Discussion

### Design and EMW Absorption Properties of Ordered Porous EDCF

**Fig. 2:**
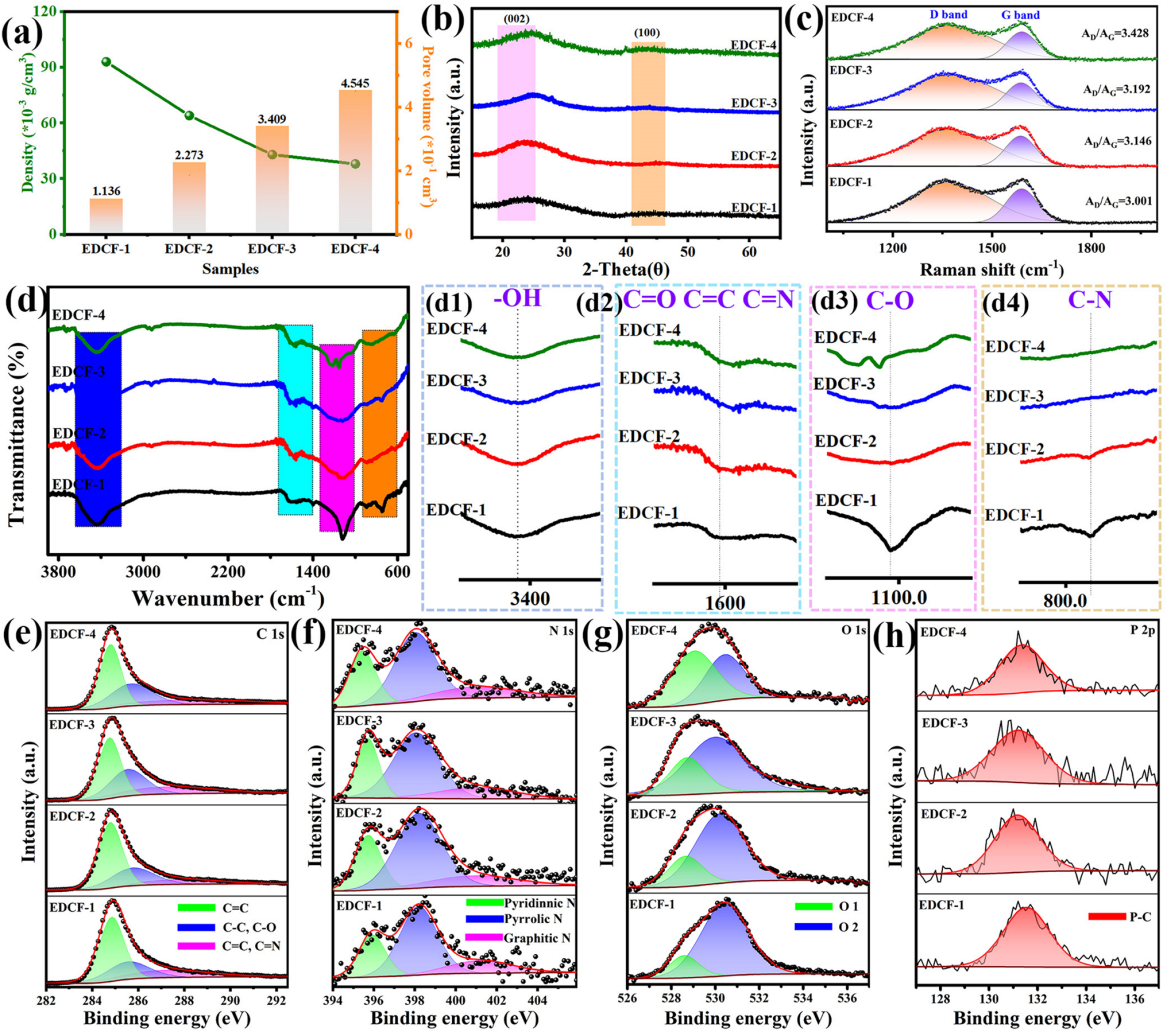
**a** Variation of density, pore volume and specific surface area of EDCF samples with different addition amount of 200 nm silica microspheres; **b** XRD patterns, **c** Raman spectra, **d** FT-IR spectra and partial enlarged images of FT-IR captured from marked area in Fig. 2d (d1 ~ d4); high-resolution XPS spectra of **e** C 1*s*, **f** N 1*s*, **g** O 1*s* and **h** P 2*p* of EDCF-1 ~ EDCF-4 samples

Figure 1b displays a typical SEM image of EDC. Obviously, the surface of the as-synthesized sample is smooth without pores or fluctuation in the absence of silica participation. Figures 1c and S1 show the SEM images of egg-derived carbon embedded with silica template microspheres (EDC@SiO_2_ nanocomposites), and a large number of silica microspheres with the diameter of about 200 nm have been evenly dispersed in the sample. To explore the effect of silica microsphere addition amount on the morphology evolution of EDCF samples, the SEM images of various samples were shown in Fig. 1d–g. Compared with the origin EDC and EDC@SiO_2_ samples, the silica microspheres were completely removed, leaving a large number of uniform spherical pores with the diameter of about 200 nm, which is consistent with the size of silica. Clearly, as the amount of silica microsphere addition increases, the created pore amount in EDCF samples also gradually increases, and the skeleton proportion (the enclosed region constructed by adjacent spherical pores, as shown in Fig. S2) gradually decreases. Since the employed silica has the same diameter, it can be inferred that the created specific surface area and pore volume of EDCF-2, EDCF-3, and EDCF-4 are 2, 3, and 4 times higher than that of EDCF-1, respectively (Tables S1 and S2). Besides, the density of the obtained EDCF samples also gradually decreases, as exhibited in Fig. 2a. Due to the delicate wall structure and high porosity, the density value of EDCF-1, EDCF-2, EDCF-3, and EDCF-4 is as low as 0.093, 0.064, 0.043, and 0.038 g cm^−3^, accordingly, thus being able to rest on the petals easily, showing an attractive advantage of ultra-lightweight character, as shown in Fig. 1h. Interestingly, some mesopores with the diameter of about 40 nm appear at the bottom of the created pore (as shown by yellow arrow) that increases gradually from EDCF-1 to EDCF-4 in quantity, which can result from the increasing packing density of silica microspheres as the increased addition amount of silica microspheres. Due to mesoporous structure, EDCF samples can constitute a penetrative network structure, which is not only favorable for removing the deeply embedded silica microspheres but also provides some additional channels for the EMW to enter the internal samples besides the created pores [33, 34].

To further explore the microstructure of EDCF samples, TEM characterization was implemented, and the typical results were revealed in Fig. 1i–k. Based on Fig. 1i, the dense accumulated pores mould the sample to form an ordered network structure. According to the aggregation-induced charge transport theory, the EDCF with the porous conductive network not only provides the capacity for multiple reflection and scattering but also enhances the conduction loss [35]. Obviously, the pores located in different layers stack together and lead to different lightness and shade contrast in the vertical direction, while the bright area corresponds to the created pore with good light transmittance, and the dark area is assigned to the carbon skeleton region with poor light transmittance. Figure 1j shows a partial enlargement image of the pores in different layers, captured from the marked area in Fig. 1i, and can be depicted by dotted circles in different colors. Figure 1k displays a typical HRTEM image of the constructed carbon skeleton, in which the lattice fringes were not recorded, indicating that the as-obtained carbon was in amorphous state [36]. However, it is necessary to note that some nano-crystalline graphite in the form of vortex/onion-like structure can be detected (as shown in the circle), which is of great importance for improving the conductivity and conduction loss of carbonaceous absorbing materials [37].

To determine the phase composition of the obtained EDCF samples, the XRD analysis was conducted, and the corresponding patterns were presented in Fig. 2b. Two broad diffraction peaks located at 26° and 43° could be assigned to the characteristic peaks of (002) and (100) crystal planes of carbon, which originates from amorphous carbon and graphite respectively. In addition to that, the broad contour and low intensity manifest that the existing main phase of EDCF samples is amorphous carbon, which is consistent with the TEM results of Fig. 1k [38]. Moreover, Raman spectra of various EDCF samples were recorded to further investigate the microstructure information of carbon components, as displayed in Fig. 2c. Then, it can be found that all of the four samples display broad D-band and G-band located around 1300 and 1580 cm^−1^, which is caused by defects and disorder structure of the materials and in-plane stretching vibration of carbon atom *sp*^2^ hybrid respectively [39]. It is well known that high-temperature heat treatment plays a crucial role in the graphitization degree, and the EDCF samples prepared at the same temperature achieve similar graphitization degree. According to the integral area values of D band and G band, the graphitization degree can be evaluated by the *A*_D_/*A*_G_ values and it is 3.001, 3.146, 3.192, and 3.428 respectively, indicating their main amorphous state [40, 41]. The slight increase tendency may be aroused by the numerous intrinsic N, O, etc. hetero-atom induced defects within carbon structure in the form of asymmetric oxygen-containing groups, which is conducive to the interfacial polarization and dipole polarization [42]. It is worth mentioning that the presence of amorphous carbon prevents the electron movement, and results in a poor conduction loss.

Figure 2d is the FT-IR spectra recorded from various EDCF samples with different addition amount of 200 nm silica microspheres, and Fig. 2d1–d4 correspond to the partial enlarged images of each characteristic peak appearing in various wavenumber segments, respectively. It can be seen that the curve tendency of FT-IR spectra is similar to each other without significant change, and the location of corresponding characteristic peaks is basically unchanged, revealing that the types of functional groups adhered to the surface of EDCF samples are not affected by the increased pore. Besides, the absorption peak at approximately 3400 cm^−1^ corresponds to the adsorbed hydroxyl (–OH) that may come from adsorbed water of the sample [43]. Multiple peaks at around 1600 cm^−1^ can be assigned to the characteristic peak of C=O, C=C, and C=N, while the peak at about 1100 cm^−1^ may be caused by C–O bond, and that near 800 cm^−1^ is mainly attributed to the stretching vibration of C–N bond [44–47]. These polar functional groups are considered as the dipole polarization center, which can induce the turning-direction polarization, thus enhancing the polarization loss and EMW absorption capacity of EDCF [48]. From the curve fitting results shown in Fig. 2d1, it can be observed that the integral area of the –OH absorption peak gradually increases from EDCF-1 to EDCF-4. Generally, these –OH groups are mainly adsorbed on the spherical surface of pores. The larger the specific surface area is, the more –OH groups are adhered to the surface. Different from the –OH group existing on the surface, C=O, C=C, C=N, and C–O, C–N bonds are mainly originated from the combination of N, O hetero-atoms in the carbon structure, which is the main cause of defect dipole polarization. Furthermore, the N and O atoms are evenly distributed in the mixed egg liquid, and their content is in inverse proportion to the total pore volume. With the increase of the created pore, the amount of C=O, C=C, C=N and C–O, C–N gradually decreases, which may weaken the dipole polarization of EDCF.

The chemical bonds and electron state of the four EDCF samples were characterized by XPS, and the corresponding results were shown in Fig. 2e–h. Figure 2e displays the C 1*s* spectra, which can be decomposed into three independent peaks at 284.6, 285.3, and 286.3 eV, corresponding to C–C/C=C, C=N, and C=O, respectively [49]. C=N and C=O bonds are beneficial to the improvement of the conductivity of the material, leading to excellent conduction loss and enhancing the EMW absorption performance of EDCF samples. Interestingly, the corresponding integral area of C–C/C=C bonds remains unchanged, while that of the C=N and C=O bonds in the EDCF samples decreases with the enhancement of the pore number. As exhibited in Fig. 2f, N 1*s* spectra of EDCF samples can be divided into three integral peaks, located at 398.3, 400.6, and 403.7 eV separately, which represent pyridine type N, pyridine type N and oxidized N [50]. It is worth noting that the relative intensity of the pyridine nitrogen peak (or the intensity ratio of pyridine nitrogen to pyrrole nitrogen) gradually decreases as the created pore increases, which is in line with the change of C=N and C–N bonds of FT-IR spectra in Fig. 2d2. Figure 2g that displays the O 1*s* peak, which can be divided into two stripping peaks appearing at 528.6 and 530.7 eV, corresponding to defect oxygen and surface absorbed oxygen, respectively [51]. Besides, the amount of the defect oxygen decreases with the increase of pore numbers, while the amount of surface absorbed oxygen in EDCF samples increases gradually. EDCF-4 achieves the largest specific surface area and pore volume, suggesting its least carbon skeleton volume. Therefore, the maximum of adsorbed –OH groups and the minimum of N, O-containing bonds can be simultaneously accomplished. Moreover, Fig. 2h displays the P 2*p* spectra of phosphorus elements corresponding to the P–C bond, whose relative intensity slightly weakens with the increased pore. In particular, various N and O atom doping in the carbon conductive networks brings a large number of internal defects and promotes the movement of electrons, generating some dipole polarization loss under an applied electromagnetic filed.

**Fig. 3:**
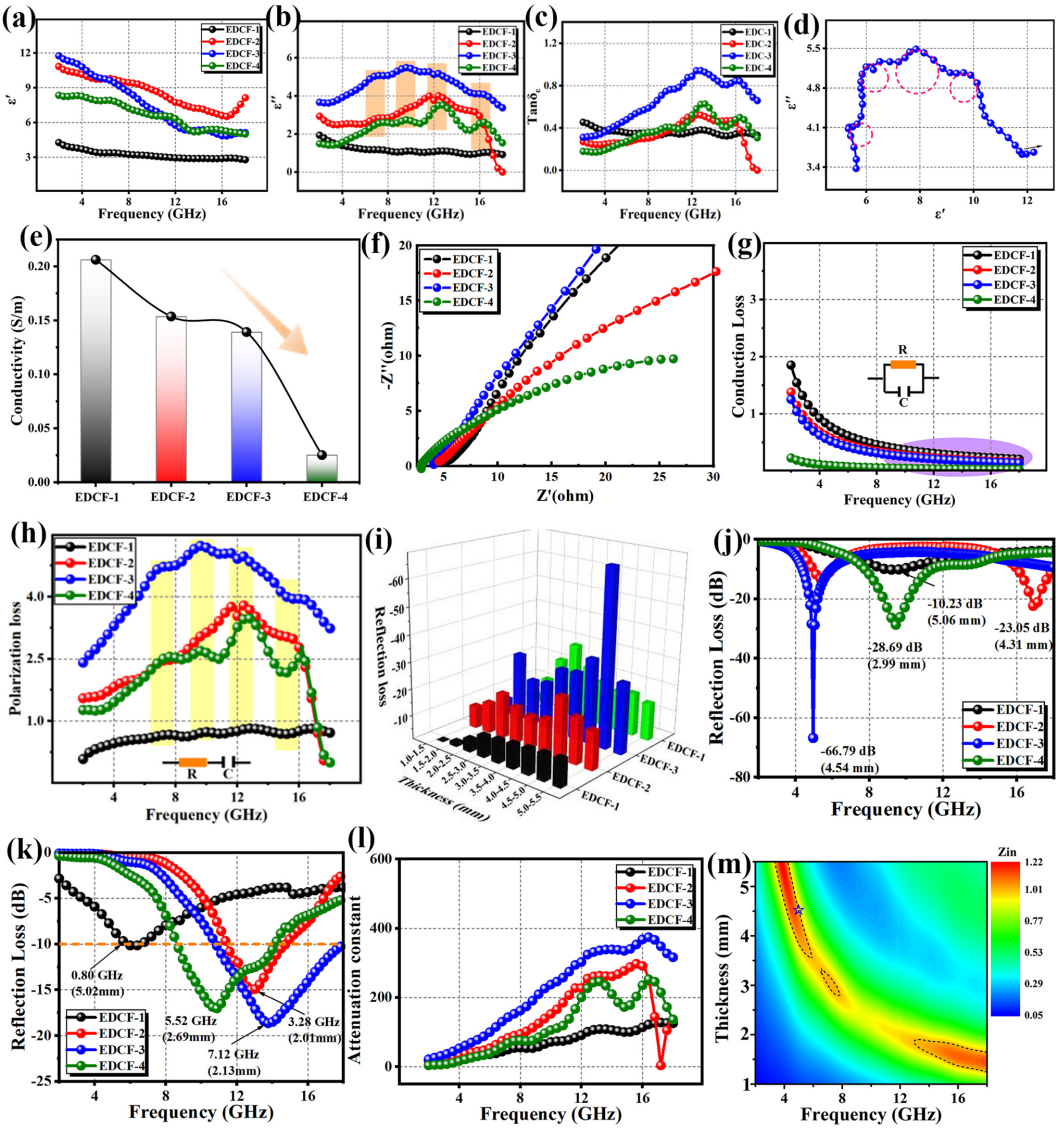
**a** The real part and **b** the imaginary part of the complex permittivity for various EDCF samples; **c** tangent of dielectric loss of the EDCF samples; **d** Cole–Cole curve of EDCF-3; **e** conductivity; **f** EIS spectrum of EDCF samples; **g** conduction loss values and **h** polarization loss values of various EDCF samples versus frequency;** i** comparison of the RL values of various EDCF samples at different thickness; **j** comparison of the minimum RL curves and **k** the widest EAB curves of different EDCF samples at their respective optimum thickness; **l** attenuation constant of EDCF samples; **m** impedance matching of EDCF-3

To evaluate the EMW absorption performances of the EDCF samples, it is necessity to investigate the variation of electromagnetic parameters. Carbon is usually considered as a dielectric loss absorbing material because of its negligible magnetic loss and complex permeability. As can be seen from the curves of the real part ($\mu '$) and imaginary part ($\mu ''$) of permeability in Fig. S3a, b, all samples are featured with the similar permeability value, and synchronous variation is kept with the change of frequency. It is speculated that the slight fluctuation phenomenon may be concerned with the magnetic moment originated from disordered carbon structure (internal hetero-atom induced defects). However, the values of $\mu '$ and $\mu ''$ stabilize near 1 and 0 respectively, indicating that the magnetic loss can be reasonably neglected [52]. In this work, the real part ($\varepsilon '$) and imaginary parts ($\varepsilon ''$) of the permittivity of EDCF samples are what we care about, which represent the electric field energy storage and attenuation ability of an absorber. In addition, the complex permittivity (*ε*_*r*_) of all samples in the frequency range of 2 ~ 18 GHz was captured at room temperature, which can be represented by the following equations [53, 54]:

1 $$\varepsilon _{r} = \varepsilon _{\infty } + \frac{{\varepsilon _{s} - \varepsilon _{\infty } }}{{1 + j2\pi f\tau }} = \varepsilon ' - j\varepsilon ''$$

2 $$\varepsilon ' = \varepsilon_{\infty } + \frac{{\varepsilon_{s} - \varepsilon_{\infty } }}{{1 + (2\pi f)^{2} \tau^{2} }}$$

3 $$\varepsilon '' = \frac{{2\pi f\tau (\varepsilon_{s} - \varepsilon_{\infty } )}}{{1 + (2\pi f)^{2} \tau^{2} }}$$

where *ε*_∞_ is the optical dielectric constant; *ε*_*s*_ refers to the static dielectric constant; *τ* denotes the relaxation time, and *f* indicates the frequency. Figure 3a, b shows the variation curves of $\varepsilon '$ and $\varepsilon '$ versus frequency in the frequency range of 2 ~ 18 GHz. Obviously, the complex permittivity of EDCF samples can be regulated by adjusting the addition amount of silica. The $\varepsilon '$ values of EDCF samples gradually decrease with the increase of the frequency, which can be attributed to the hysteresis of dielectric polarization caused by the variation of electromagnetic field (chromatic dispersion) [55]. Noteworthily, the smallest $\varepsilon '$ value appears in the EDCF-1 with the minimum number of pores, and the $\varepsilon '$ curves of EDCF-2 display the largest values. In the low-frequency range, the $\varepsilon '$ value of EDCF-3 is larger than that of EDCF-4, but both values change synchronously with the increase of frequency in the middle and high-frequency range. As exhibited in Fig. 3b, EDCF-1 has the smallest $\varepsilon ''$ value, and EDCF-3 achieves the largest $\varepsilon ''$ value, indicating its strongest EMW attenuation capacity. Other than that, EDCF-2 and EDCF-4 have roughly the same $\varepsilon ''$ values. Furthermore, Fig. 3c shows the dielectric loss (tan*δ*_*ε*_ = $\varepsilon '$/$\varepsilon ''$) curves. It can be found that the variation of tan *δ*_*ε*_ is more dependent on the imaginary part of permittivity, and in the meanwhile, EDCF-3 acquires the largest value in the measured frequency, which changes more sharply than that of the other three samples, revealing its excellent high-frequency response. As shown in Fig. S3c, the magnetic loss tangent (tan*δ*_*μ*_ = $\mu '$/$\mu ''$) values are always equal to 0, suggesting that no magnetic loss was exerted on the EMW attenuation for EDCF samples. Moreover, the Cole–Cole curve can effectively demonstrate the polarization relaxation phenomenon of the absorber, and each semicircle in the curve corresponds to a Debye relaxation process. The value can be calculated by the following equations [56, 57]:

4 $$\left( {\varepsilon ' - \frac{{\varepsilon_{s} + \varepsilon_{\infty } }}{2}} \right)^{2} + (\varepsilon '' )^{2} = \left( {\frac{{\varepsilon_{2} - \varepsilon_{\infty } }}{2}} \right)^{2}$$

Figures 3d and S4 exhibit the Cole–Cole curves of EDCF samples. It can be determined that all of the EDCF samples display several semicircles marked in red colour, suggesting that multiple Debye relaxation phenomena occur, which can effectively dissipate the energy of the incident EMW [58]. In addition, EDCF-2 produces the most Debye dipole relaxation process, resulting in the largest ε' value, which is consistent with Fig. 3a. Generally, the dielectric loss and ε' curve variation of the absorber are mainly influenced by dipole polarization relaxation and interfacial polarization. With the increase of pores, the surface of EDCF produces more dangling bonds/absorbed functional groups and few N, O, and P hetero-atoms, leading to strong interfacial polarization and weak defect-induced dipole polarization. To some extent, the $\varepsilon '$ value can be considered as the result of the competition between the interfacial polarization and the internal dipole polarization of the products. Noteworthily, previous works have pointed out that the slop of the linear end of Cole–Cole curves represents the conduction loss of the absorbing materials. Interestingly, the slope of Cole–Cole captured from EDCF samples gradually decreases with the increase of the pore number, implying their decreased conduction loss [59].

It is well known that the dielectric loss of the absorbing materials can be classified into conductance loss and polarization loss. For conduction loss, the value is closely associated with dielectric conductivity. As the graphitization degree is typically dominated by high treatment temperature, the EDCF samples prepared at the same temperature obtain similar graphitization degree and electron migration capability, meaning that the conduction loss of the EDCF sample is weak [60, 61]. For polarization loss, the main contribution can be ascribed to interface polarization and dipole polarization, because the electron/ion displacement polarization phenomenon doesn’t seem to have an impact on ε_r_. In order to clarify the source of dielectric loss of the EDCF samples, the conduction loss and polarization loss should be analyzed in detail, which can be measured according to the following equation [62, 63]:

5 $$\varepsilon '' = \varepsilon_{p} '' + \varepsilon_{c} '' = (\varepsilon_{s} - \varepsilon_{\infty } )\frac{2\pi f\tau }{{1 + (2\pi f)^{2} \tau^{2} }} + \frac{\sigma }{{2\pi f\varepsilon_{0} }}$$

where $\varepsilon _{p} ''$ represents the contribution of polarization loss; $\varepsilon_{c} ''$ is the contribution of conduction loss; *σ* refers to the dielectric conductivity, and *ε*_0_ (*ε*_0_ = 8.854 × 10^–12^ F m^−1^) denotes the vacuum permittivity. The theoretical fitting calculation values of σ for various EDCF samples were shown in Fig. 3e, which exhibits opposite varying tendency with the pore volume, consistent with the EIS spectrum (Fig. 3f) [64]. In fact, the porous EDCF samples can be served as a complex made up of solid medium and spherical pores. Moreover, in this work, a larger number of holes mean higher resistivity (poor conductivity). Subsequently, the conduction loss and polarization loss curves were shown in Fig. 3g, h. In addition, the equivalent circuit can be regarded as the parallel and series circuit of resistance and capacitor, respectively [65, 66]. In Fig. 3g, the conduction loss curves display the same decrease trend with the increasing frequency, and the corresponding values reach 0.2 ~ 0.3 in the high-frequency range. The polarization loss was also calculated and exhibited in Fig. 3h, and the variation tendency and the frequency of polarization loss peaks of the corresponding results were very close to the resonance of $\varepsilon ''$ curves, which is also in well accord with the Debye relaxation phenomenon. Furthermore, it can be found that polarization loss is slightly larger than conduction loss in the frequency range of about 2 ~ 8 GHz, but reaches one order of magnitude higher than the value of conduction loss in the frequency range of about 8 ~ 18 GHz. The dielectric loss originated from the synergistic effect of conduction loss and polarization loss in S-band and C band. However, the contribution of conduction loss with very small value can be neglected in X band and Ku band, which is mainly provided by polarization loss. As demonstrated in Fig. 3i, their relationship can be briefly described as the unity of opposites.

According to transmission line theory, the reflection loss (RL) value can be calculated by the following equations [19, 67, 68]:

6 $$Z_{{{\text{in}}}} = Z_{0} \sqrt {\frac{{\mu_{r} }}{{\varepsilon_{r} }}\tanh \left[ {j\left( {\frac{2\pi fd}{c}} \right)\sqrt {\mu_{r} \varepsilon_{r} } } \right]}$$

7 $${\text{RL}}\;({\text{dB}}) = 20\log \left| {\tfrac{{Z_{{{\text{in}}}} - Z_{0} }}{{Z_{{{\text{in}}}} + Z_{0} }}} \right|$$

where *Z*_0_ refers to the impedance of free space; *Z*_in_ represents the input impedance; *ε*_*r*_ (*ε*_*r*_ = $\varepsilon '$ − *j*$\varepsilon ''$) means the complex permittivity; *μ*_*r*_ (*μ*_*r*_ = $\mu '$ − *j*$\mu ''$) denotes the complex permeability; *f* signifies the frequency of the input EMW; *d* is the thickness, and *c* represents the light velocity. Fig. S5 shows the RL curves of various EDCF samples in the frequency range of 2 ~ 18 GHz at different matching thicknesses. Obviously, the located frequency of the minimum reflection loss value (RL_min_) for EDCF samples displays a shift towards lower frequency with the increase of corresponding thickness. In addition, EDCF-1 presents poor EMW absorption performance with the RL_min_ of − 10.23 dB at 5.06 mm thickness, and its effective absorption bandwidth (EAB, < − 10 dB) is exceedingly narrow. For EDCF-2, EDCF-3, and EDCF-4 samples, the RL and EAB can be effectively changed by adjusting the thickness in the frequency range of 4 ~ 18 GHz. Compared with other samples, the EDCF-3 gains excellent EMW absorption performances with the RL_min_ of − 66.79 dB at the thickness of 4.54 mm. Meanwhile, it achieves the widest EAB of 7.12 GHz at 2.13 mm thickness. When the matching thickness is greater than 4 mm, EDCF-1 and EDCF-4 capture two RL_min_ peaks in C band and Ku band, respectively. In addition, with the increases of the pore number, the located frequency of the RL_min_ shifts towards low frequency, while the EAB value is mainly concentrated in the X-band and Ku band. The attenuation constant (*α*) of the samples was measured, which is strongly associated with the dissipation ability of electromagnetic waves [69].

8 $$\alpha = \frac{{\sqrt 2 \pi f}}{c}\sqrt {\left( {\mu ''\varepsilon '' - \mu '\varepsilon '} \right) + \sqrt {\left( {\mu ''\varepsilon '' - \mu '\varepsilon '} \right)^{2} + \left( {\mu '\varepsilon '' + \mu ''\varepsilon '} \right)^{2} } }$$

Figure 3m shows the *α* values of various EDCF samples. Obviously, the curves display a similar sequence of samples as Fig. 3c (EDCF-3 > EDCF-2 > EDCF-4 > EDCF-1) in the frequency range of 2 ~ 18 GHz, suggesting the dominant effect of dielectric loss on the α values. Impedance matching is an important indicator for evaluating the EMW entering the material, which can be evaluated by *Z*_in_ value according to Eq. 6. Ideally, when the input impedance matching of the absorbing material is equal to the free space impedance, it is implied that the EMW was introduced completely. Figures 3n and S6 show the *Z*_in_ contour chart of EDCF samples at different frequencies and thicknesses. It can be seen that the value of EDCF-3 fluctuates slightly around *Z*_in_ = 1, indicating its good impedance matching, and the EMW can enter the sample effectively, which is also a prerequisite for achieving excellent EMW absorption performances. Moreover, Fig. S7 presents the normalized impedance matching characteristic of EDCF samples. Then, it can be found that better RL_min_ and EAB values appear in the EDCF-3 with *Z*_in_ value approaching 1 although the α value at the frequency of RL_min_ is far less than that of other samples, revealing that the decisive effect of impedance matching on EMW absorption performances.

**Fig. 4:**
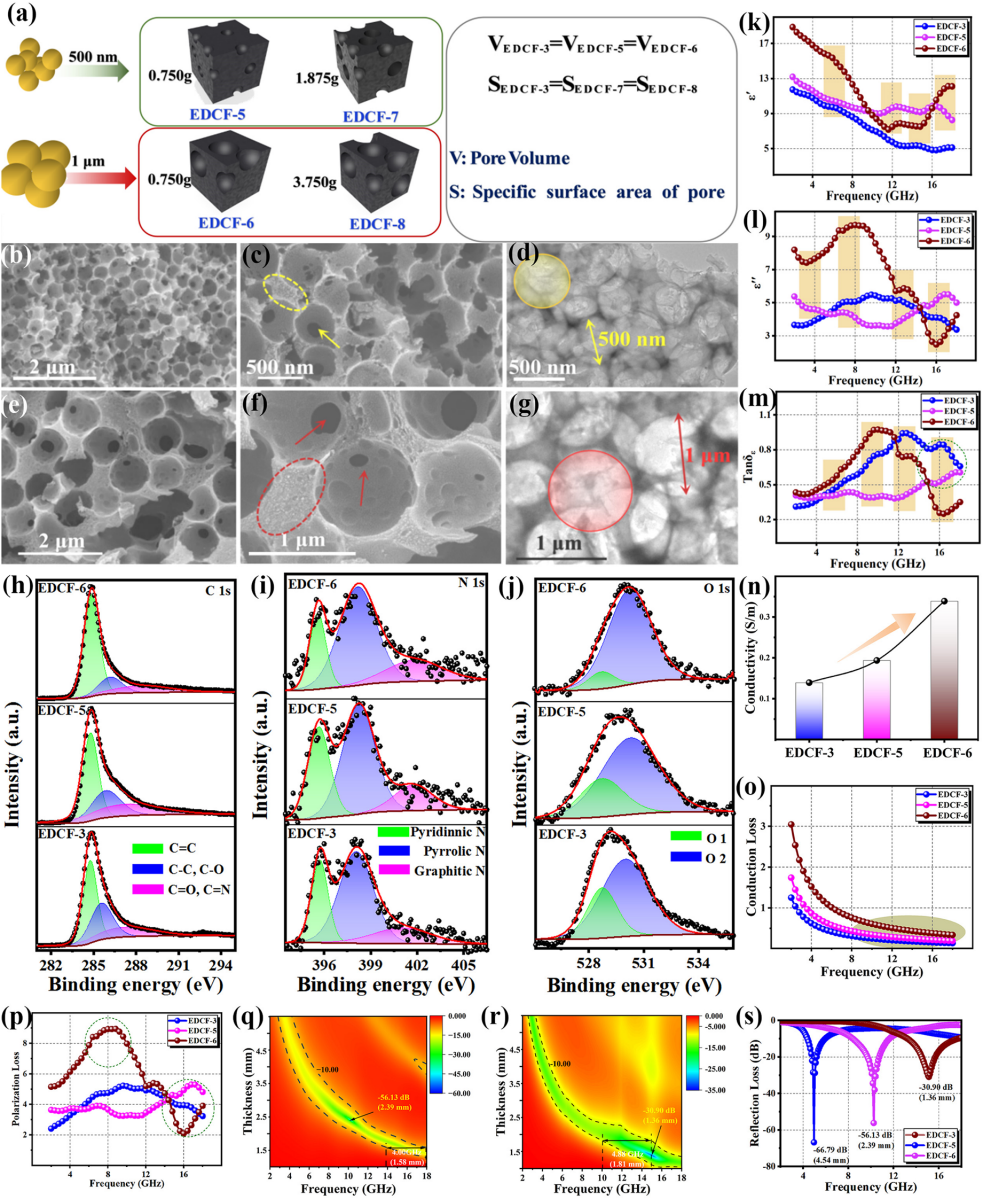
**a** Schematic diagram of EDCF-5 and EDCF-7 constructed using 500 nm silica microspheres, and EDCF-6 and EDCF-8 constructed using 1 μm silica microspheres; **b**, **c** SEM images and **d** TEM image of EDCF-5 sample; **e**, **f** SEM images and **g** TEM image of EDCF-6 sample; high-resolution XPS spectra of **h** C 1*s*, **i** N 1*s*, and **j** O 1*s* of EDCF-3, EDCF-5 and EDCF-6 samples; **k** the real part and **l** the imaginary part of the complex permittivity and **m** dielectric loss tangent for EDCF samples with the same pore volume; **n** conductivity values; **o** conduction loss curves and **p** polarization loss curves of EDCF-3, EDCF-5 and EDCF-6 samples; **q** RL diagram versus frequency of EDCF-5 and r EDCF-6 at different thickness; **s** comparison of the RL values of various EDCF samples at their optimal thickness

To clarify the influence of pore volume on the electromagnetic parameters and EMW absorption performances, it is necessary to ensure a single variable, that is, avoiding the interference of and specific surface area. Therefore, two EDCF samples (EDCF-5 and EDCF-6) were constructed under the assist of silica microspheres with the diameter of 500 nm and 1 μm respectively (as shown in Fig. 4a), and the addition content was fixed at 0.75 g, which is consistent with EDCF-3. Obviously, EDCF-3, EDCF-5, and EDCF-6 are featured with the same pore volume (*V*_EDCF-3_ = *V*_EDCF-5_ = *V*_EDCF-6_) but different specific surface areas (*S*_EDCF-3_ > *S*_EDCF-5_ > *S*_EDCF-6_). A large number of uniform pores with the diameter of 500 nm equivalent to the employed silica microsphere template have been inserted into the carbon skeleton of EDCF (as shown in Fig. S8). Figure 4b, c are the SEM images of EDCF-5, and it can be observed that some mesoporous structures similar to EDCF-3 were formed at the bottom of the pore. Figure 4d shows a typical TEM image of EDCF-5. Obviously, a large number of pores with the diameter of 500 nm in different layers accumulate successively and fill into the as-prepared product. Besides, no obvious lattice structure is detected at the discernible edge position, indicating that the obtained carbon material exists in amorphous state. Figures 4e, f exhibit the SEM images of EDCF-6, and the diameter of the created pore is increased to 1 μm while a larger silica microsphere template is used. On the one hand, as the diameter of the employed silica microsphere increases, the number of pores significantly decreases (as depicted in the field of vision), whereas, on the other hand, the volume of an individual enclosed carbon skeleton area (as shown by the red arrow) is accordingly increased. Moreover, in Fig. 4g, a typical TEM image of EDCF-6 appears, revealing a uniform pore diameter of about 1 μm, and there is still abundant space for accommodating more silica microspheres.

Figure S9 displays the XRD pattern and Raman spectra captured from the EDCF samples with the same pore volume (EDCF-3, EDCF-5, and EDCF-6), respectively. Clearly, all of the three EDCF samples display the same phase and microstructure, indicating that the composition was not affected by the size of created pores. As shown in Fig. S10, due to the same volume proportion of carbon skeletons, the relative intensity of carbon-containing bonds located at 800, 1100, and 1600 cm^−1^ keeps unchanged, while the characteristic peak at around 3400 cm^−1^ assigned to the adsorbed –OH group is featured with a reduced relative intensity from EDCF-3 to EDCF-5 and EDCF-6 with the decrease of specific surface area. Figure 4h, j displays the high-resolution XPS spectra of C 1*s*, N 1*s*, and O 1*s* recorded from EDCF-3, EDCF-5, and EDCF-6, accordingly. In C 1*s* spectra (Fig. 4h), the bonding energy, relative intensity and the integral area of each divided peak from the three EDCF samples are roughly identical. In general, nitrogen atoms are mainly distributed in the carbon skeleton, and its relative content is in inverse proportion to the pore volume. Obviously, the integral area ratios of pyridinic N to pyrrole N, and pyridinic N to graphite N of above three EDCF samples are roughly identical, demonstrating their same amount. In Fig. 4j, the relative intensity and integral area of peak O1 decrease while those of peak O2 keep constant, implying that the smaller the created pore diameter (higher specific surface area), the more functional group adsorbed on the surface when the EDCF samples have the same pore volume. Furthermore, the interfacial polarization generated by the adsorbed functional group will be less for EDCF-6 compared with EDCF-3 and EDCF-5.

Figure 4k, l present the variation curves of the $\varepsilon '$ and $\varepsilon ''$ values captured from the three samples as a function of frequency. Clearly, the $\varepsilon '$ values of EDCF-5 and EDCF-6 are larger than that of EDCF-3 in most measured frequencies, and EDCF-5 displays a similar variation tendency to that of EDCF-3 with the increase of frequency. The $\varepsilon '$ curve of EDCF-3 and EDCF-5 decreases gradually, while that of EDCF-6 decreases first and then increases when the maximum value is obtained in the frequency range of 2 ~ 10 and 16 ~ 18 GHz, indicating its good storage capacity of electric field energy. The maximum $\varepsilon '$ value was occupied by EDCF-5 in the frequency range of 10 ~ 16 GHz. As can be seen from Fig. 4l, the $\varepsilon ''$ values of EDCF-5 slightly fluctuate between 3 and 5 by adjusting the measured frequency. For EDCF-6, the $\varepsilon ''$ values reach the maximum of 9.70 at 8 GHz, and then rapidly decrease to the minimum value at 16 GHz followed by a slight increase. In the medium and high-frequency range, EDCF-6 has larger values than EDCF-3 and EDCF-5, revealing its excellent dielectric loss capacity. However, lower values are collected in the high-frequency range. It is well known that these maximum or minimum values generally can be regarded as resonance peaks, which is corresponding to different polarization or relaxation processes. Interestingly, EDCF-3 possesses the same carbon skeleton and larger specific surface area as EDCF-5 and EDCF-6, suggesting that EDCF-3 may produce more interfacial polarization. Nevertheless, EDCF-6 is given the larger value of complex permittivity when compared with EDCF-3. It can be speculated that the value may be related to the diameter of pores. Figure 4m shows the curve of tan*δ*_*ε*_ values with frequency variation, and the overall tendency of each sample is similar to the curves in Fig. 4l, revealing the stronger dielectric loss capacity of EDCF-3 and EDCF-6 for the incident EMW. In the medium and low-frequency ranges of 2 ~ 12 GHz, the largest tan*δ*_*ε*_ value of EDCF-6 appears. However, EDCF-3 achieves the largest tan*δ*_*ε*_ values in the high-frequency range of 12 ~ 18 GHz.

Figure S11 shows the Cole–Cole curves of EDCF samples with the same pore volume. It can be discovered that all of the three samples have multiple relaxation processes, and the appearing frequency of Debye relaxation marked by dotted circles almost corresponds to the frequency of resonance peaks of $\varepsilon ''$ and $\varepsilon '$. The dielectric conductivity was calculated and exhibited in Fig. 4n, and the values increase gradually with the increase of silica size and specific surface area. Accordingly, as shown in Fig. 4o, the EDCF-6 sample with 1000 nm pore diameter achieves higher conductive loss, which is generally consistent with the tail slope of Cole–Cole curves and may provide some reference for analyzing the conductive loss of absorbing materials. It can be speculated that when the EDCF samples were endowed with the same pore volume, the smaller the specific surface area is, the fewer surface adsorption functional groups it contains, and the electron quenching phenomenon or the number of bound electrons will also be reduced, which effectively enhances the conductivity and increases the conduction loss of the sample. At the same time, as shown in Fig. 4p, the polarization loss of EDCF-6 increases gradually in the range of 4 ~ 8 GHz, and reaches the maximum value at around 8 GHz. Subsequently, it decreases sharply in the range of 8 ~ 16 GHz. Therefore, it can be determined that the three EDCF samples contain the same pore volume (fraction of carbon skeleton), so it can be assumed that the number of hetero-atoms is approximately identical, suggesting that the contribution of conduction loss and defect-induced dipole polarization to EMW attenuation can be cancelled out for them [70]. Furthermore, the difference in EMW absorption performances for these contrast samples can be attributed to the interfacial polarization originated from the surface adsorbed functional group.

Figure 4q, r depicts the 2D projection drawing of EDCF-5 and EDCF-6 in the frequency range of 2 ~ 18 GHz at various thicknesses. With the increase of thickness, the appearing frequency of the RL_min_ shifts towards lower frequency. Compared with EDCF-6, EDCF-3 (Fig. 3j), and EDCF-5 obtain a wider frequency range with the value below − 10 dB by adjusting the thickness. As presented in Fig. 4s, the corresponding RL_min_ value of the three samples at the optimal thickness of them is summarized. Obviously, EDCF-3 also possesses the best RL_min_ values. Meanwhile, the matching thickness of EDCF-5 and EDCF-6 is thinner than that of EDCF-3, whereas the widest EAB of EDCF-5 and EDCF-6 appears at the thickness of 1.58 and 1.81 mm respectively, with the corresponding value of 4 GHz (14 ~ 18 GHz) and 4.88 GHz (10 ~ 15.68 GHz), which is narrower than that of EDCF-3. As displayed in Fig. S12, EDCF-5 obtains the RL_min_ value of -56.13 dB appearing in X-band at the thickness of 2.39 mm, and the RL_min_ of EDCF-6 is − 30.90 dB at 1.36 mm thickness achieving Ku-band. Apparently, the located frequency of RL_min_ can be effectively adjusted by changing the pore diameter of different EDCF samples, and it shifts towards high frequency with the increase of pore diameter. Meanwhile, the RL_min_ value and corresponding matching thickness gradually decreases with the decrease of specific surface area (increase of pore diameter) when the pore volume of the EDCF samples is fixed. Figure S13a, b illustrates the impedance matching and attenuation constant curves of the EDCF-5 and EDCF-6 samples. It can be found that the *Z*_in_ value of them is closer to 1 just in the high-frequency range, which is consistent with the located frequency of RL_min_, manifesting their good impedance matching. Moreover, it should be noted that the variation of specific surface area nearly had no effect on the impedance matching when the pore volume was optimized. As shown in Fig. S13c, d, EDCF-3 obtains the highest α value in the best impedance matching region. Therefore, it also achieves the best EMW absorption performances.

### Equivalent Specific Surface Area Substitution and EMW Absorption Properties of EDCF

**Fig. 5:**
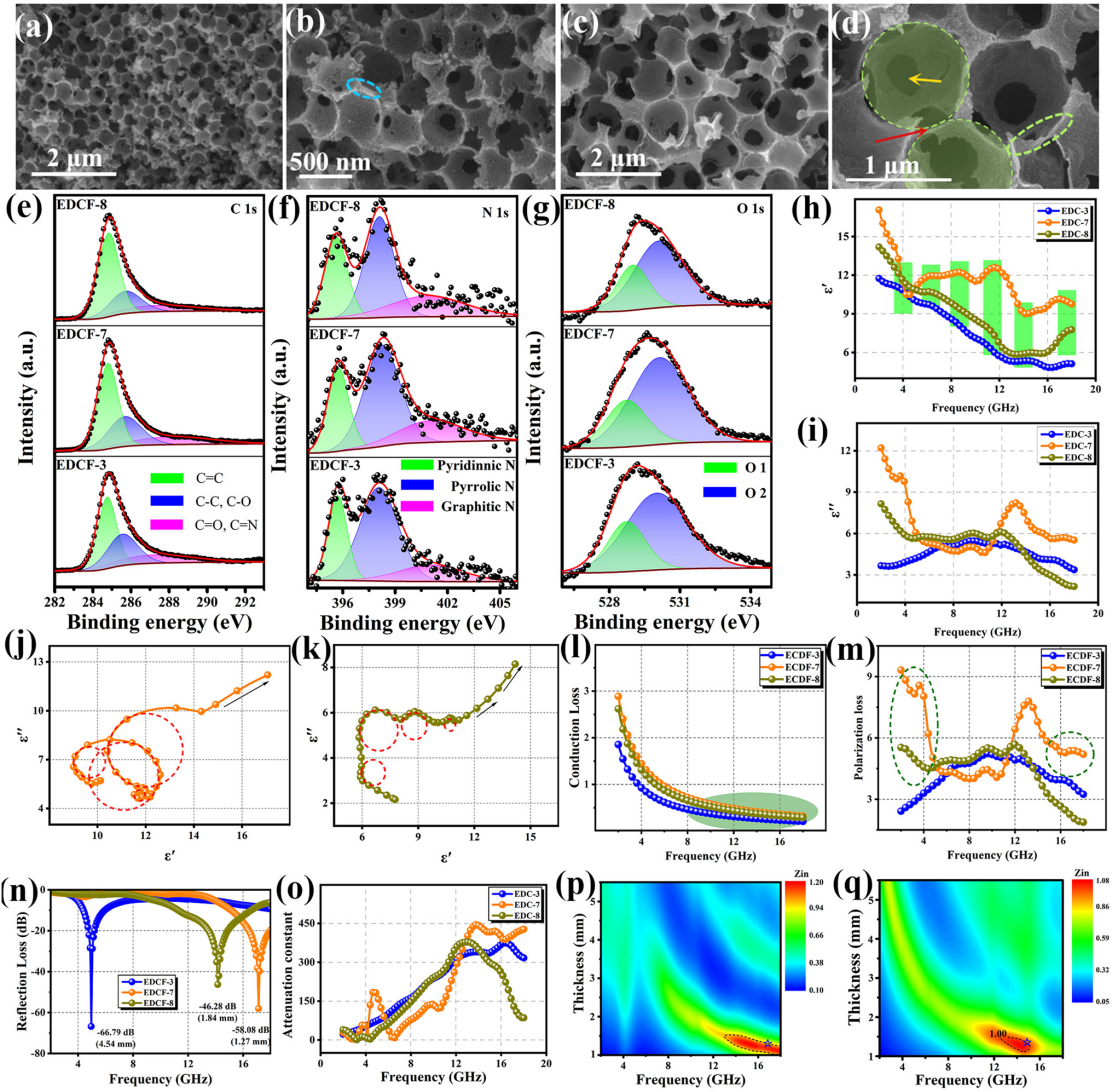
**a, b** SEM images of EDCF-7 sample; **c**, **d** SEM images of EDCF-8 sample; high-resolution XPS spectra of **e** C1*s*, **f** N1*s*, and **g** O1*s* of EDCF-3, EDCF-7 and EDCF-8 samples; **h** the real part and **i** the imaginary part of the complex permittivity; **j** Cole–Cole curves of EDCF-7 and **k** EDCF-8; **l** conduction loss and **m** polarization loss of EDCF samples; **n** comparison of the minimum RL curves for EDCF samples with the same specific surface area; **o** attenuation constant and impedance matching of **p** EDCF-7 and **q** EDCF-8

In order to investigate the influence of specific surface area on the EMW absorption performances of EDCF samples, two contrast samples, EDCF-7 and EDCF-8 with the same specific surface area and different pore volumes as EDCF-3 were tactfully constructed by introducing silica microspheres with the diameters of 500 nm and 1 μm respectively (as demonstrated in Fig. 4a). The addition content of silica microspheres with the diameter of 500 nm is 1.875 g, while the dosage of silica microspheres with the diameter of 1 μm employed to produce EDCF-8 is approximately 3.75 g. It is believed that EDCF-3, EDCF-7, and EDCF-8 acquire the approximately equivalent adsorbed functional groups, and further stimulate the same surface polarization. In addition, what is different about the EDCF samples with the same specific surface area is their pore volume (*V*_EDCF-3_ < *V*_EDCF-7_ < *V*_EDCF-8_, *S*_EDCF-3_ = *S*_EDCF-7_ = *S*_EDCF-8_), implying that the content of hetero-atoms decreases from EDCF-3 to EDCF-7 and EDCF-8. Therefore, the difference in EMW absorption performances for these samples can be mainly attributed to the volume proportion of carbon skeleton-related defect-induced dipole polarization. Figure S14 exhibits the SEM images of DC@SiO_2_ samples with the same specific surface area, whereas Fig. 5a, b is SEM images of EDCF-7. In this case, it can be observed that numerous pores with the diameter of 500 nm have been created after removing embedded silica microspheres. Moreover, Fig. 5c, d exhibits the SEM images of EDCF-8 with the pore diameter of 1 μm distributed uniformly. Noteworthily, compared with EDCF-5 and EDCF-6 with the same pore diameter, the constructed carbon skeleton of EDCF-7 and EDCF-8 also decreased to a great extent.

Figure S15 displays the XRD pattern and Raman spectra captured from the EDCF samples with the same specific surface area, respectively. Obviously, all of the three EDCF samples feature the same phase and microstructure, indicating that the composition was not affected by the volume or diameter of pores. Besides, their FT-IR spectra were shown in Fig. S16, in which the intensity and integral area of absorption peaks are hardly changed, implying their same type and content of surface-absorbed functional groups. Figure 5e–g are XPS spectra of C, N, and O elements captured from EDCF-3, EDCF-7, and EDCF-8 samples. In Fig. 5e, it can be observed that the integral area of C–C/C–O band and C=O/C=N bond slightly decreases with the increase of pore volume. Meanwhile, as displayed in Fig. 5f, although the relative intensity of the N-containing peak decreases gradually, the integral area ratio of pyridine nitrogen, pyrrole nitrogen and graphite nitrogen of various EDCF samples does not change dramatically, implying that the content of nitrogen atoms is decreasing by the same proportion aroused by the volume increase of pores. Furthermore, Fig. 5g exhibits the O 1*s* spectra of EDCF samples, and it can be found that the relative intensity of the O1 peak keeps constant, while the O2 peak decreases with the increase of pore volume.

Figure 5h, i display the $\varepsilon '$ and $\varepsilon ''$ curves of the EDCF samples with the same specific surface area. The $\varepsilon '$ curves of the three samples show a decreasing trend versus frequency, and the $\varepsilon ''$ curves of the three samples show a completely opposite trend in the range of low frequency (2 ~ 6 GHz), high frequency (12 ~ 18 GHz) and middle frequency (6 ~ 12 GHz). With the other samples, there are multiple resonance phenomena in the curve, indicating the multiple polarization relaxation process occurs [71]. With the same specific surface area, the three samples obtain the same surface defects and adsorption functional groups, that is, the produced interface polarization effect is roughly equivalent. In other words, the higher permittivity values of EDCF-7 than EDCF-8 and EDCF-3 can result from its stronger defect-induced dipole polarization. Besides, the curve of dielectric loss tangent values with frequency variation for EDCF-7 and EDCF-8 was shown in Fig. S17a. Compared with EDCF-3, the curves can be divided into three sections. In the frequency range of 2 ~ 6 GHz, the tan*δ*_*ε*_ curve of EDCF-7 obtains larger values than EDCF-3 and EDCF-8; in the frequency range of 6 ~ 12 GHz, EDCF-7 displays the smallest values, while the change of EDCF-3 and EDCF-8 is almost synchronous in the frequency range of 12 ~ 18 GHz. All of them decrease sharply, and EDCF-3 still has the largest values. Furthermore, in Fig. 5j, k, the Cole–Cole curves of EDCF-7 and EDCF-8 appear respectively, and it can be found that EDCF-7 has more Debye relaxation processes than EDCF-3 and EDCF-8, which may acquire stronger polarization loss. The big slope of the linear tail suggests the high conductivity of EDCF-7. According to free electron theory, the conductivity from various samples was shown in Fig. S17b, and EDCF-7 also achieves the highest conductivity value. The conduction loss and polarization loss were calculated respectively, and the corresponding results were shown in Fig. 5l, m. Clearly, the order concerning conduction loss of EDCF samples is the same as that of conductivity, and the polarization loss value versus frequency is similar to *ε*" curves. In the frequency range of 2 ~ 6 GHz and 12 ~ 18 GHz, EDCF-7 possesses the highest values, while it obtains the smallest values in the frequency range of 6 ~ 12 GHz.

Figure S18 shows the contour chart of EDCF-7 and EDCF-8 samples in the frequency of 2 ~ 18 GHz at different thickness. Obviously, the located frequency of the RL_min_ shows a migrating trend towards lower frequency with the increase of corresponding matching thickness. For sample EDCF-7, the EAB can be adjusted just in the frequency range of 8 ~ 18 GHz at different thickness. Meanwhile, EDCF-8 is characterized by the broader adjustable frequency range crossing almost 2 ~ 18 GHz and achieves better EAB. Figure 5n exhibits the comparison concerning the RL_min_ of the EDCF samples with the same specific surface area at respective optimized thickness. Compared with EDCF-3, the RL_min_ of EDCF-7 and EDCF-8 appears in Ku band, which is weaker with the values of − 58.08 and − 46.28 dB respectively whereas the corresponding matching thickness is obviously thinner than the former. Apart from that, Fig. S20a, b displays the impedance matching of EDCF-7 and EDCF-8. Obviously, their proper impedance matching occurs only at thin thickness and high frequency, which is narrower than EDCF-3. Besides, Fig. 5o shows the attenuation constant curves versus frequency, and the EDCF samples with the same specific surface area exhibit similar α values at different frequency, revealing their EMW attenuation abilities are basically the same, especially in X band and Ku band. At the same time, their nearly equal values suggest that the pore volume has little effect on the *α* value when the optimal specific surface area is fixed. Furthermore, the contour chart of the impedance matching recorded from EDCF-7 and EDCF-8 was displayed in Fig. 5p, q. It can be found that the value of *Z*_in_ slightly deviates from 1 with the increase of pore volume, suggesting that the impedance matching is mainly determined by pore volume when the influence of specific surface area is removed first.

### EMW Attenuation Mechanism of EDCF

**Fig. 6:**
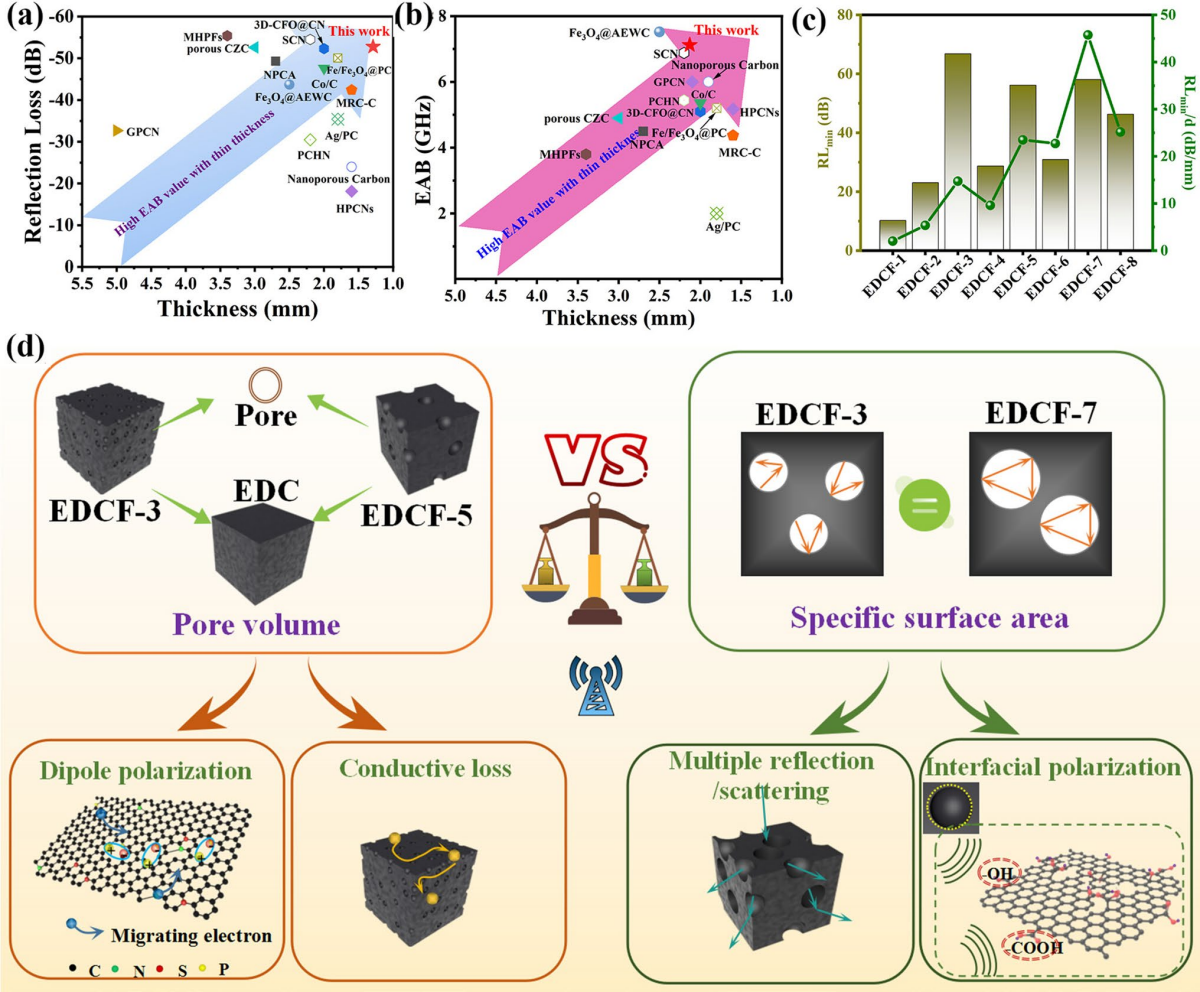
**a** RL_min_ and **b** EAB versus thickness of carbon-based absorbing materials reported recently; **c** the ratio of RL_min_ to corresponding matching thickness of various EDCF samples; **d** schematic diagram of electromagnetic attenuation strategies for EDCF samples

Figure 6a, b illustrates the RL_min_ and EAB performances of several lightweight carbon absorbers reported recently. Compared with other reported works, the obtained EDCF-7 and EDCF-3 achieve the superior RL_min_ value and EAB performance at thinner thickness, implying that the as-prepared optimal product could be regarded as an attractive candidate for the electromagnetic wave absorber. According to above characterization results, it can be concluded that the polarization loss exhibits an appropriate congruent relationship and synchronous variation tendency with the imaginary part of permittivity, revealing that the dielectric loss is mainly controlled by polarization loss. In addition, there is a very interesting phenomenon that the larger pore volume of EDCF samples with the same specific surface area, the weaker of the RL value. Meanwhile, the larger specific surface area of EDCF samples with the same pore volume, the weaker the RL value. For one thing, the increased specific surface area increases the number of adsorbed functional groups and strengthens the attenuation of incident EMWs caused by interface polarization. For another, the increased pore volume brings more internal defects, acting as the polarization center, that is conducive to dipole polarization and enhances the loss of EMWs. Ideally, the EDCF sample with large specific surface area and pore volume may possess excellent EMW absorption performances. However, it is contradictory to increase or decrease the pore volume and specific surface area at the same time. The EDCF-7 with a compromise-specific surface area and pore volume is regarded as the next-besting sample. In order to evaluate the comprehensive EMW absorption performances, the ratio of RL_min_ to corresponding matching thickness was calculated, and it should be the more feasible and concerned parameter for the industrial community, as shown in Fig. 6c. Moreover, EDCF-3, EDCF-7 and EDCF-5 take the top three spots of maximum RL values respectively, and at the same time, EDCF-7 with the value of 45.73 dB mm^−1^ acquires much better than other products. Noteworthily, compared with tedious cross experiments, the experimental design strategy employed in this work can quickly determine the optimal sample and offers a more convenient, rapid and accurate optimal scheme for screening the absorption performances.

According to the above analysis, the interfacial polarization mainly originated from the surface defects and absorbed functional groups, which is directly related to the specific surface area, and the effect of dipole polarization on the EMW absorption performances is proportional to the number of defective atoms or heteroatoms in the carbon skeleton (pore volume), indicating that the interfacial polarization and dipole polarization have opposite effects on the electromagnetic parameters of porous carbon-absorbing materials. The EMW absorption performance of the samples is influenced by the interfacial polarization and dipole polarization, which is the result of competition between interface polarization and defect-induced polarization, as shown in Fig. 6d. Besides, the main factors can be summarized as follows: The porous structure increases the interfacial area and corresponding polarization relaxation loss of samples, while the energy is easily dissipated when incident EMWs are irradiated into the porous absorber [72]. Compared with the EDC material without pores, abundant porous structure of EDCF samples can effectively reduce the complex permittivity and promote the impedance matching of the absorber according to Maxwell–Garnett (MG) theory. In this case, it is promised that more EMWs reach the interior of the absorber and expand their effective absorption band. Moreover, the ordered arrangement of pores constructs a conductive network, which not only exerts electrical loss but also is beneficial for the multiple reflection and scattering of electromagnetic waves, enhancing the capacity of converting electromagnetic energy into heat energy [73]. Furthermore, the pyridine-type nitrogen atom that was embedded in the carbon lattice structure can improve the conductivity of EDCF, which is easy to migrate and transmit electrons and dissipates the energy of electromagnetic waves through polarization relaxation. Many defects and functional groups containing N, O, and P atoms result in localized states of Fermi level, which is also conducive to the absorption and attenuation of electromagnetic waves [74]. In the solid-air interface, a large amount of charge in the external electromagnetic field is accumulated, resulting in strong space charge polarization, which plays a positive role in attenuating EMW energy [75]. In other words, the pore volume (carbon skeleton) exerts more influence on the absorption performance than the specific surface area. Appropriate pore volume provides the posterior shield for excellent absorbing performance. In conclusion, the proposed equivalent substitution strategy demonstrates that dipole polarization is the key to determining the dielectric loss behavior of EDCF.

## Conclusions

According to the principle of equivalent substitution, 3D ordered porous EDCF samples with the same specific surface area and same pore volume were respectively prepared by the template method. The results reveal that the number and diameter of pores not only decrease the density of the absorber but also influence the interfacial polarization and dipole polarization loss of the EMW. The specific surface area can improve the dipole polarization of the product for the purpose of obtaining better EMW absorbing performance. Due to the synergistic effect of interfacial loss and moderate dipole polarization, the optimal sample EDCF-7 obtains excellent RL_min_ of − 58.08 dB at the thickness of 1.27 mm. Meanwhile, EDCF-3 with proper pore structure shows the widest EAB of 7.12 GHz at the thickness of 2.13 mm. More importantly, this work covers the deficiency of the influence of specific surface area on electromagnetic properties, providing a certain reference for the structural design and mechanism research of porous EMW absorbing materials.

## Supplementary Information

Below is the link to the electronic supplementary material.

Supplementary file1 (PDF 2602 KB)
