# Supplementary material for: An Equivalent Substitute Strategy for Constructing 3D Ordered Porous Carbon Foams and Their Electromagnetic Attenuation Mechanism
Source: Nanomicro Lett. 2022 Aug 2;14:157. doi: 10.1007/s40820-022-00900-x (PMC9346049; doi:10.1007/s40820-022-00900-x)
Supplement: Supplementary file 1 — Supplementary file1 (PDF 2602 KB) [file 40820_2022_900_MOESM1_ESM.pdf]

Supporting Information for

An Equivalent Substitute Strategy for Constructing 3D Ordered Porous Carbon Foams and Their Electromagnetic Attenuation Mechanism

Meng Zhang[1], Hailong Ling[1], Ting Wang[2], Yingjing Jiang[1], Guanying Song[1], Wen Zhao[2], Laibin Zhao[1], Tingting Cheng[1], Yuxin Xie[1], Yuying Guo[1], Wenxin Zhao[1], Liying Yuan[1], Alan Meng[2], Zhenjiang Li[1,] *

[1] College of Materials Science and Engineering, College of Electromechanical Engineering, Qingdao University of Science and Technology, Qingdao 266061, P. R. China

[2] State Key Laboratory Base of Eco-chemical Engineering, College of Chemistry and Molecular Engineering, College of Chemical Engineering in Gaomi Campus, Qingdao University of Science and Technology, Qingdao 266042, P. R. China

*Corresponding author. E-mail: zhenjiangli@qust.edu.cn (Zhenjiang Li)

Supplementary Figures and Tables

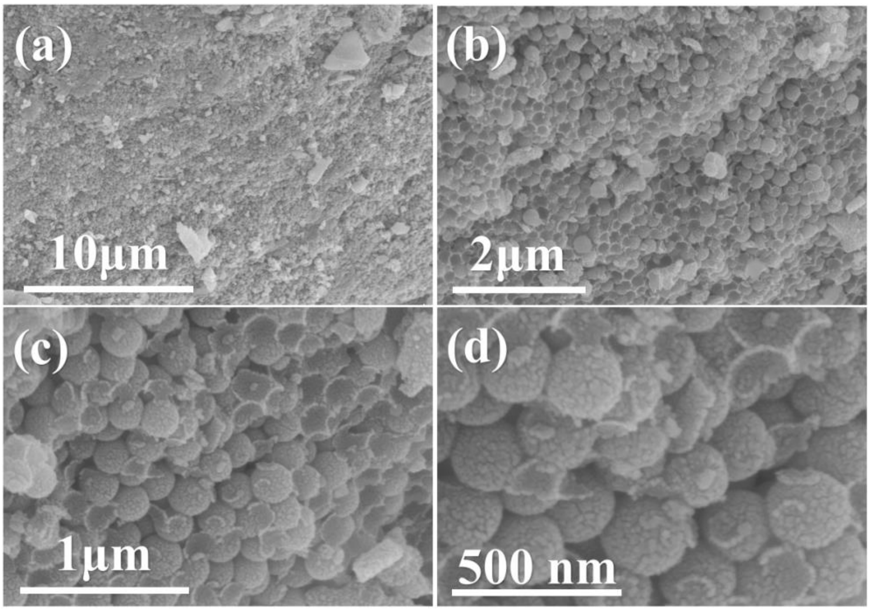

Fig. S1 SEM images of egg-derived carbon embedded uniform silica microspheres

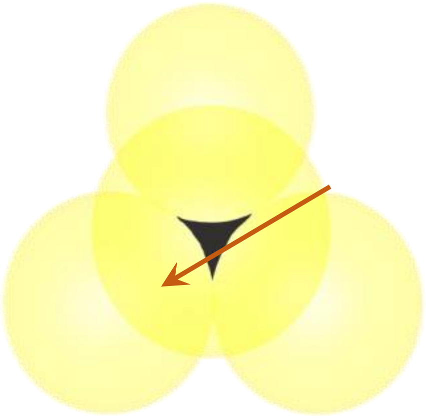

Fig. S2 Schematic image of the enclosed region constructed by adjacent four spherical pore (carbon skeleton)

Table S1 The created pore volume and specific surface area of an individual silica microspheres template with different radius.

Diameter (nm)	Radius (cm)	Density (g cm^{-3})	Volume (cm^3)	Specific surface area (cm^2)
200	$1.\ 0\times10^{-5}$		4.1888×10^{-15}	1.2566×10^{-9}
500	2.5×10^{-5}	~2.2	6.5450×10^{-14}	2.5000×10^{-9}
1000	5.0×10^{-5}		5.2360×0^{-13}	1.0000×10^{-8}

Table S2 The pore volume and specific surface area of various EDCF samples created by removing silica template

Samples	Mass (g)	Diameter of employed SiO_2 sphere (nm)	Volume of the created hole (cm^3)	Specific surface area of the created hole (cm^2)
EBCF-1	0.250	200	0.1136	34080
EBCF-2	0.500	200	0.2273	68190
EBCF-3	0.750	200	0.3409	102270
EBCF-4	1.000	200	0.4545	136350
EBCF-5	0.750	500	0.3409	40908
EBCF-6	0.750	1000	0.3409	20454
EBCF-7	1.875	500	0.8591	102270
EBCF-8	3.750	1000	1.7045	102270

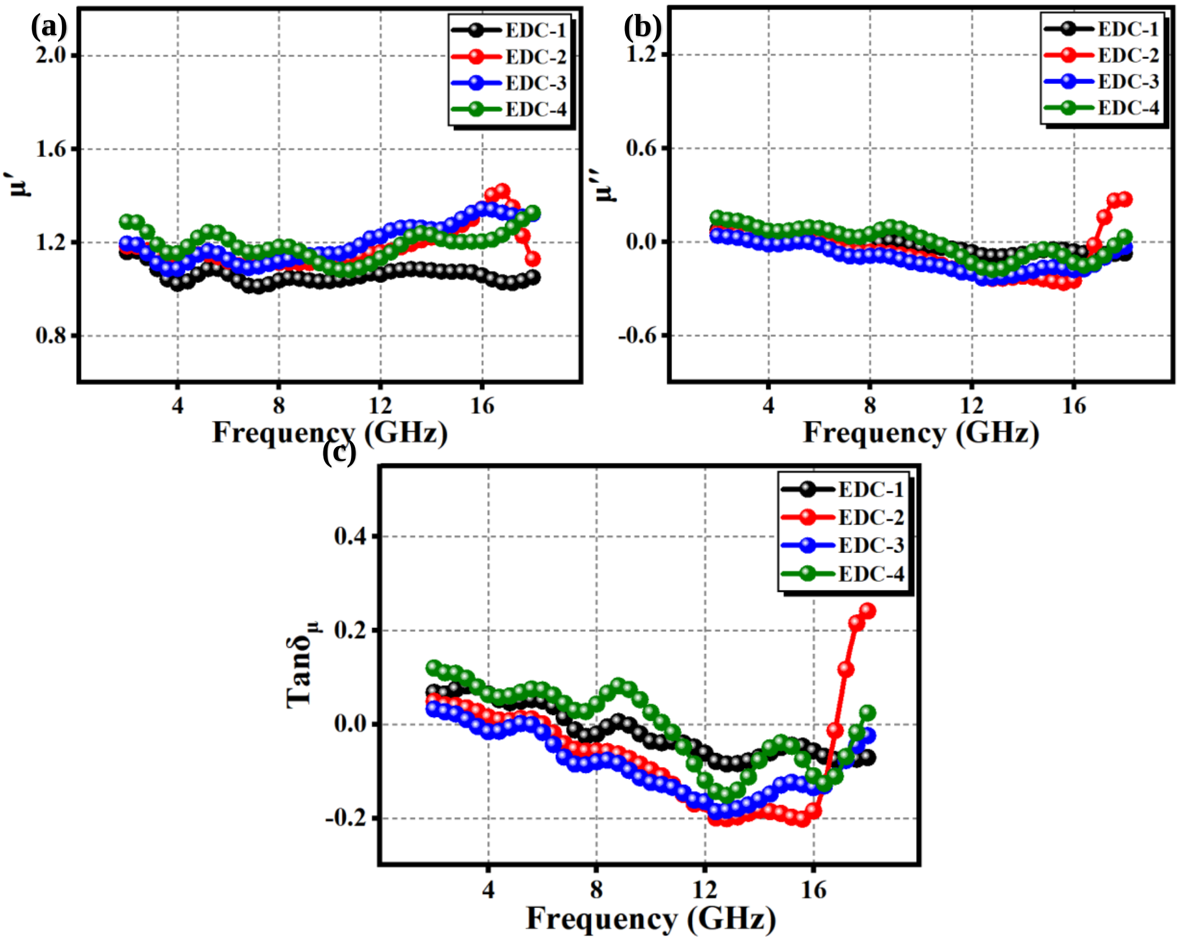

Fig. S3 The real part (**a**), the imaginary part (**b**) of the complex permeability and the magnetic loss tangent (**c**) of EDCF-1~EDCF-4 samples

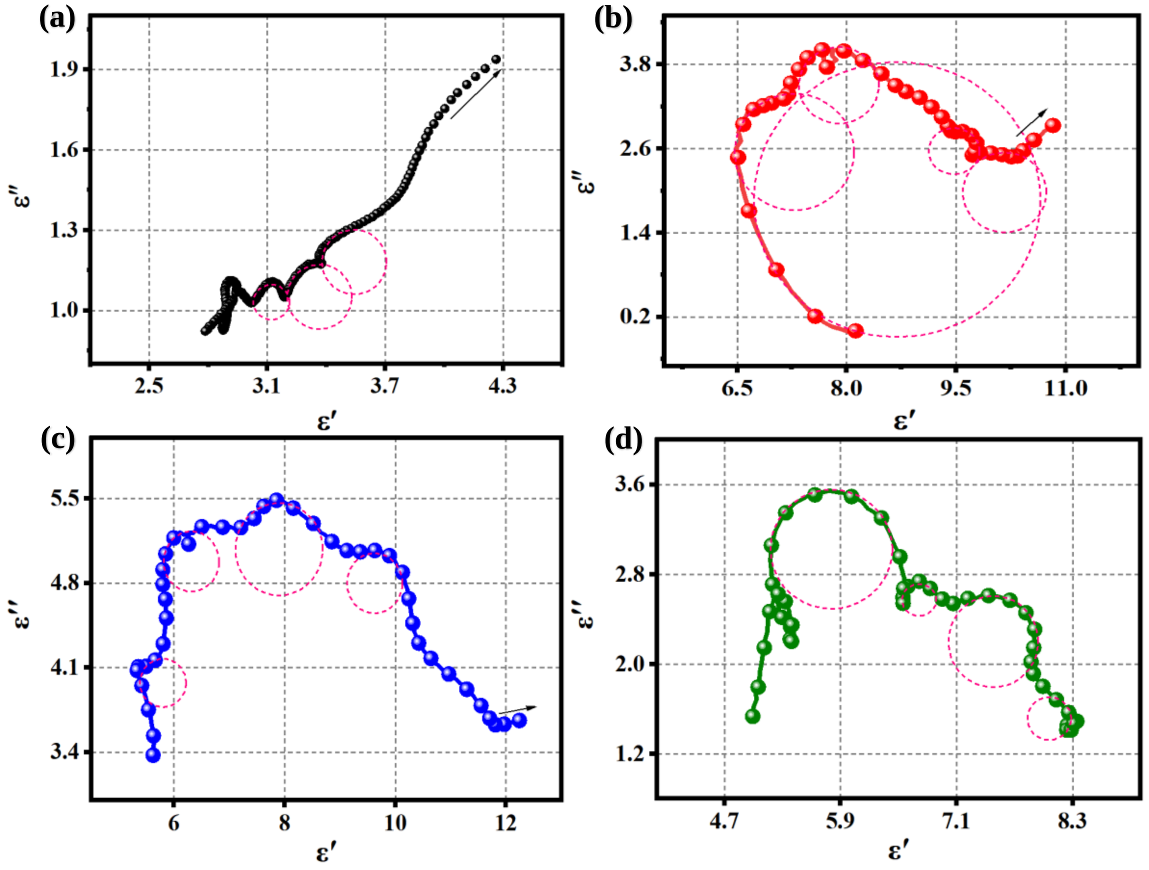

Fig. S4 Cole-Cole semicircles of EDCF-1 (**a**), EDCF-2 (**b**), EDCF-3 (**c**) and EDCF-4 (**d**)

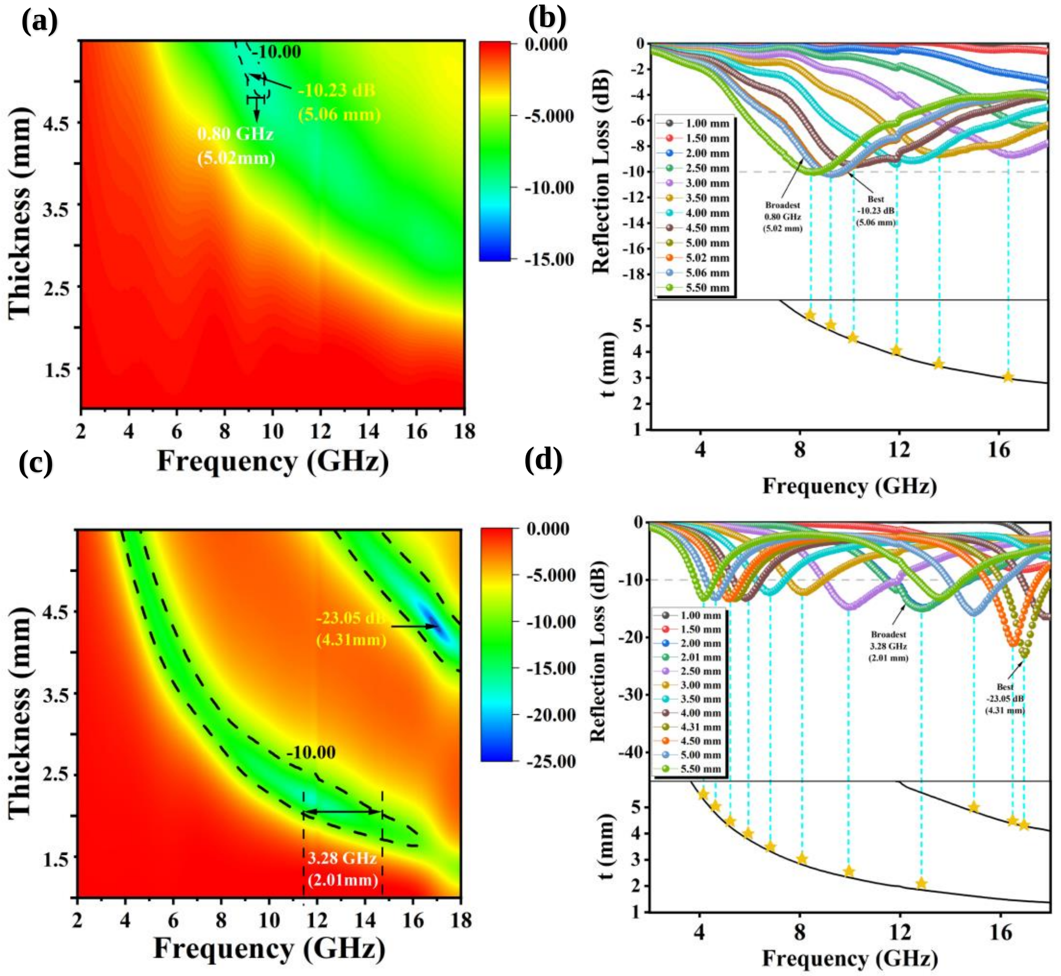

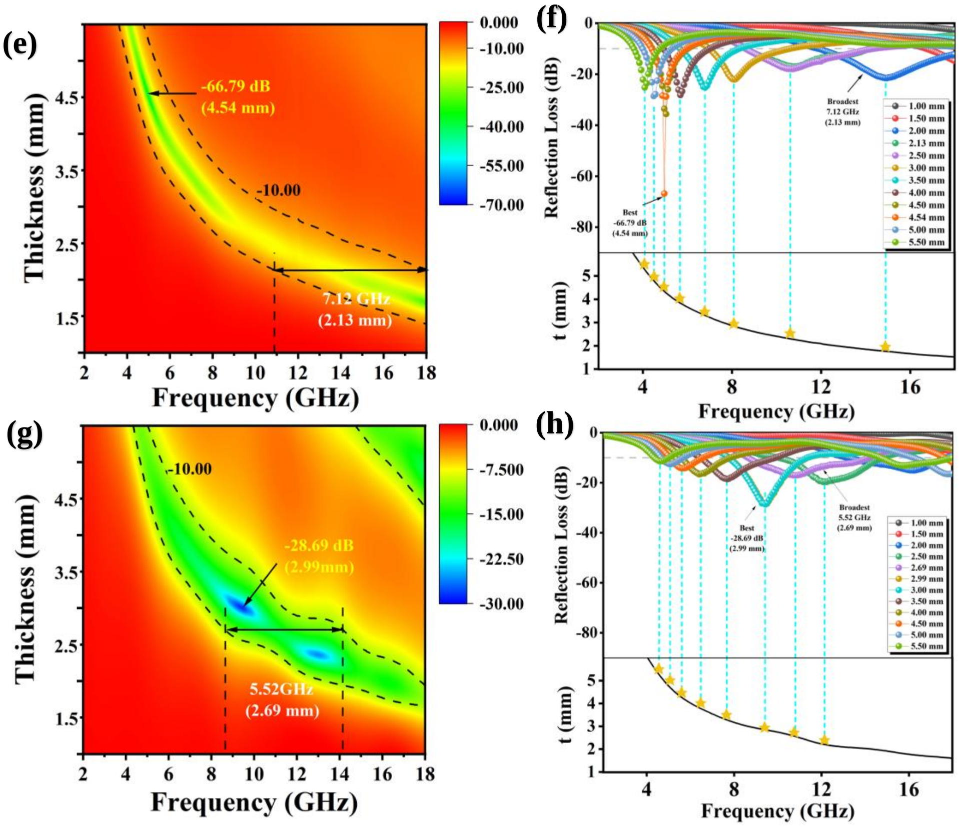

Fig. S5 Reflection loss curves versus frequency of EDCF-1 (**a, b**), EDCF-2 (**c, d**), EDCF-3 (**e, f**) and EDCF-4 (**g, h**)

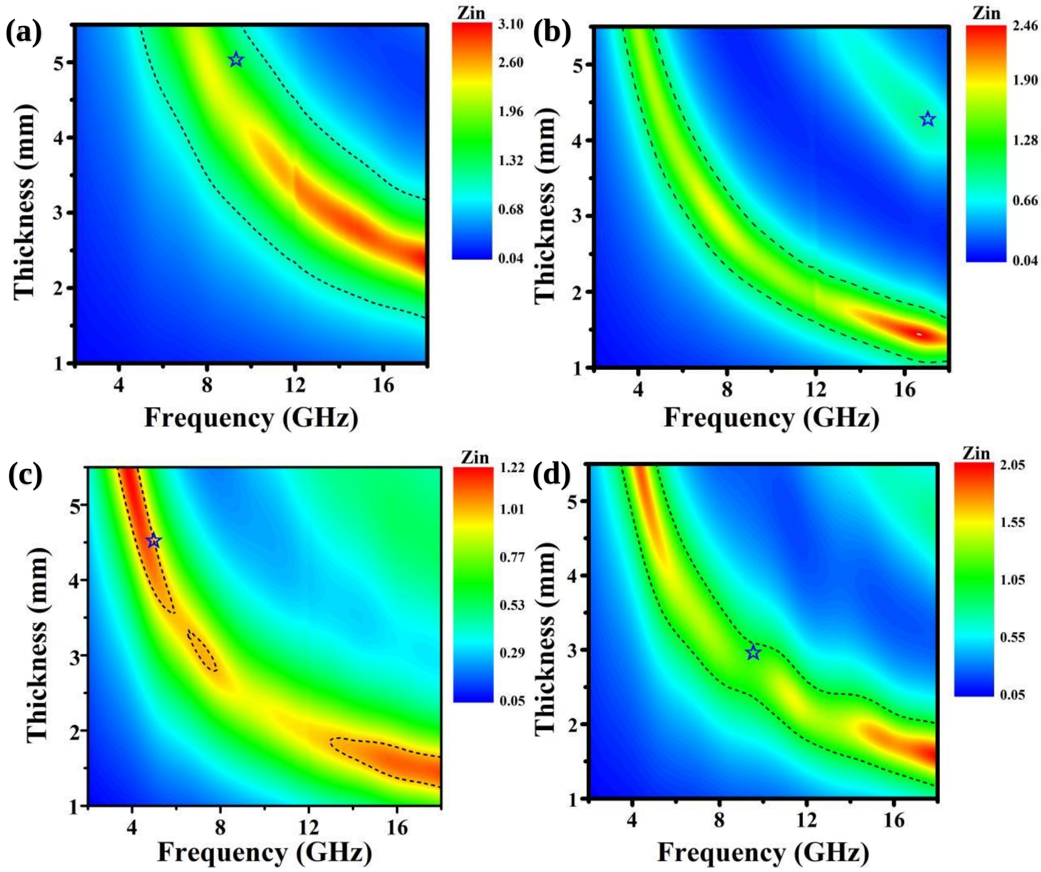

Fig. S6 Z_{in} drawing of EDCF-1 (**a**), EDCF-2 (**b**), EDCF-3 (**c**) and EDCF-4 (**d**) samples

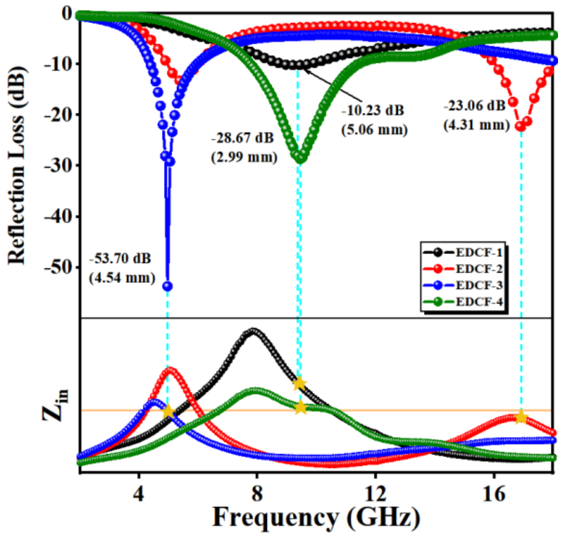

Fig. S7 Normalized impedance matching characteristic of EDCF samples

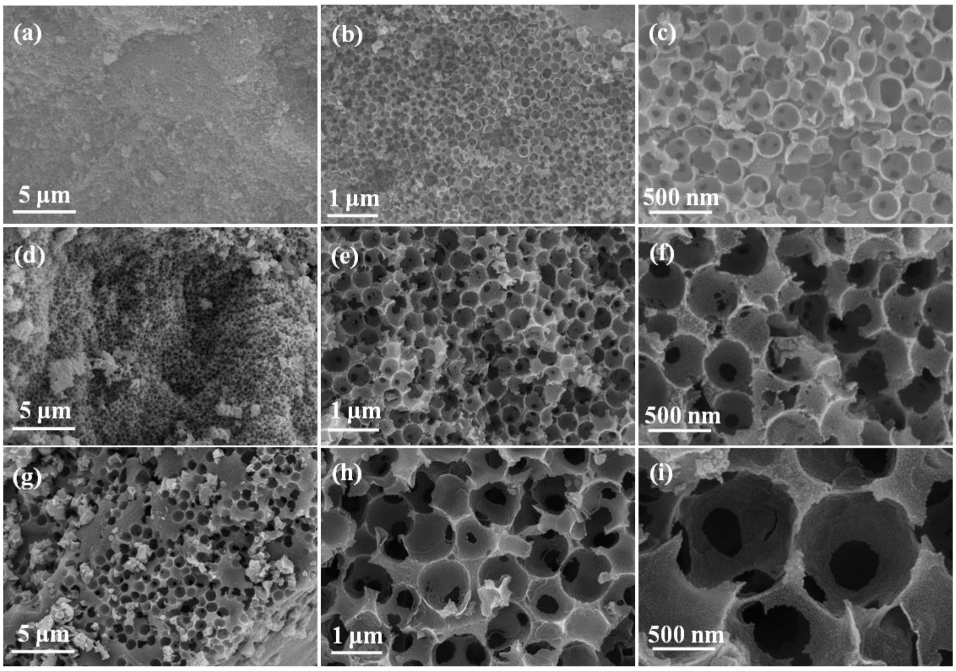

Fig. S8 EDCF samples with same volume of created hole, EDCF-3 (**a-c**), EDCF-5 (**e-f**), EDCF-6 (**g-i**)

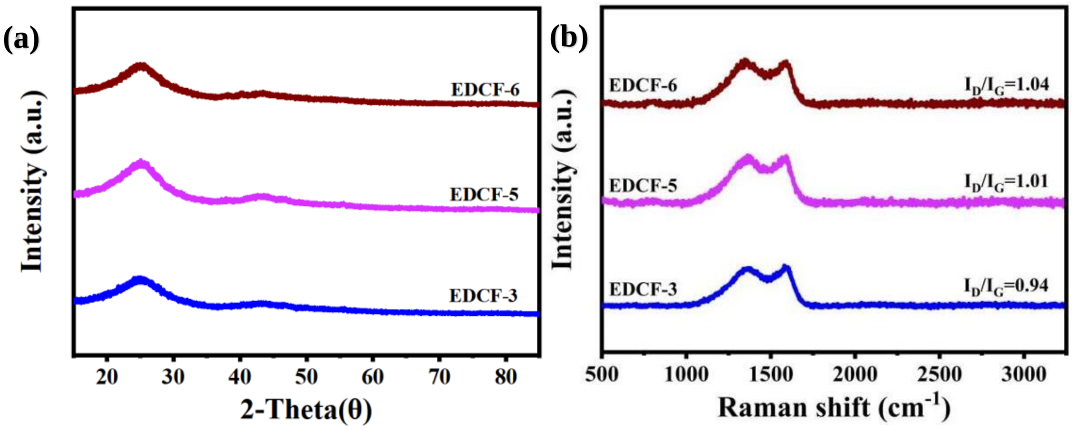

Fig. S9 XRD pattern (**a**) and Raman spectra (**b**) of various EDCF samples with same pore volume

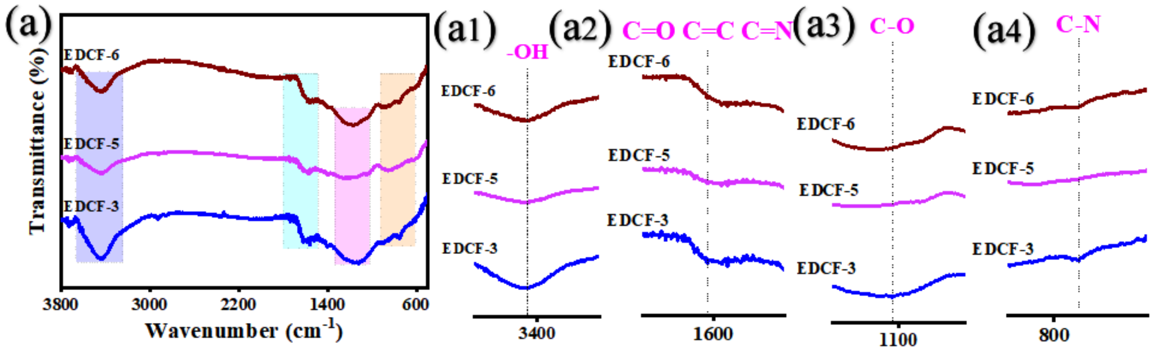

Fig. S10 FT-IR spectra of various EDCF samples with same pore volume

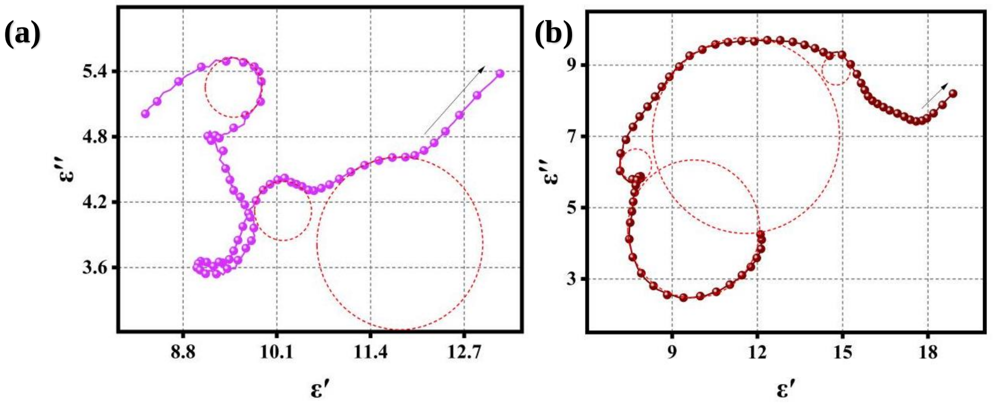

Fig. S11 Cole-Cole semicircles of EDCF-5 (**a**) and EDCF-6 (**b**)

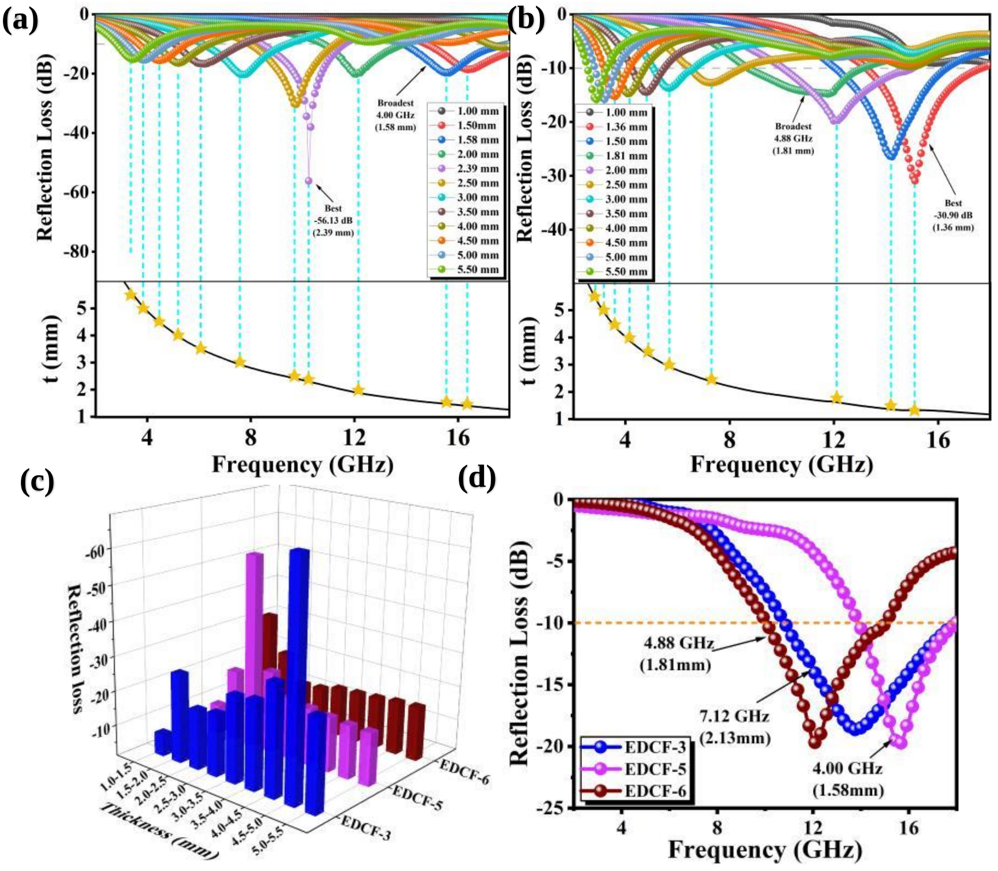

Fig. S12 RL curves of EDCF-5 (**a**), EDCF-6 (**b**) at different thicknesses of 2~18GHz; Comparison of minimum RL value of the samples at different thickness (**c**); Comparison of EAB values of EDCF-3, EDCF-5 and EDCF-6 (**d**)

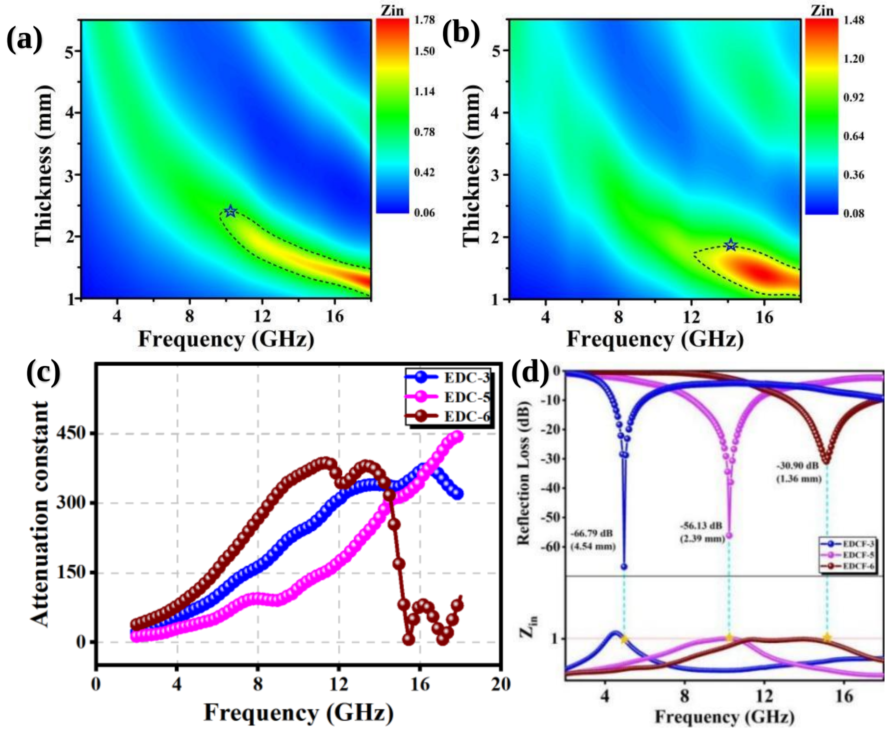

Fig. S13 Z_{in} drawing of EDCF-5 (**a**) and EDCF-6 (**b**) samples, attenuation constant (**c**) and normalized impedance matching characteristic of EDCF-3, EDCF-5 and EDCF-6 samples (**d**)

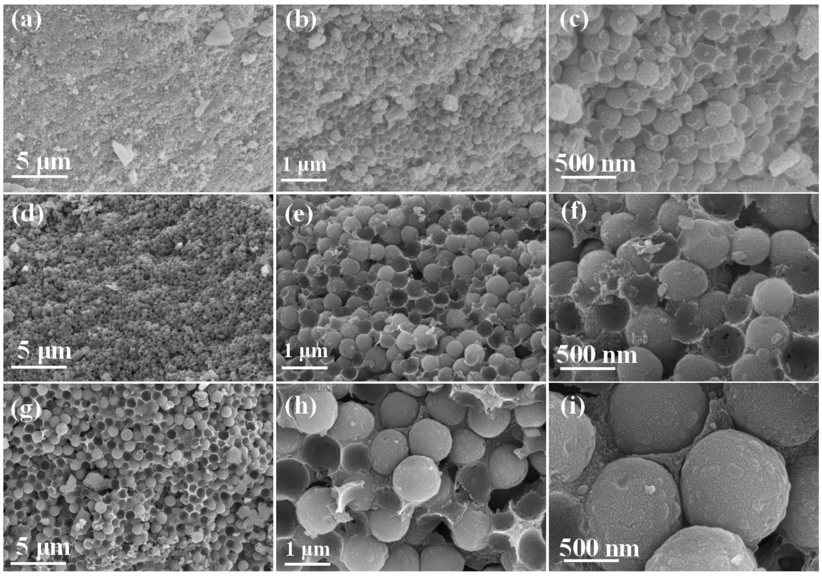

Fig. S14 EDCF samples with the same specific surface area before removing embedded silica microspheres, EDCF-3 (**a-c**), EDCF-7 (**d-f**) and EDCF-8 (**g-i**)

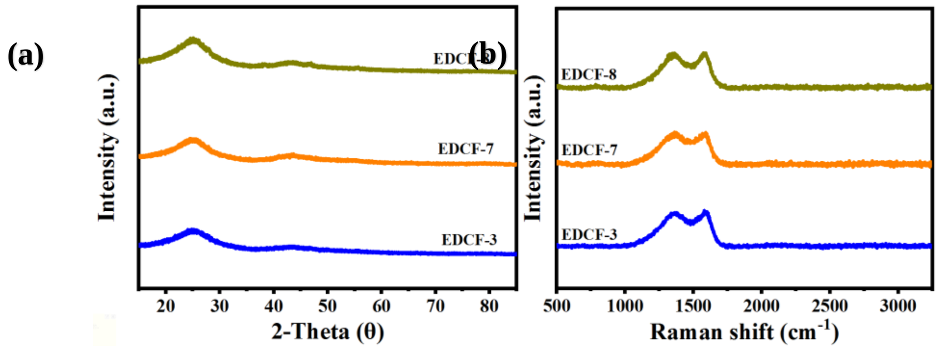

Fig. S15 XRD pattern (**a**) and Raman spectra (**b**) of various EDCF samples with same specific surface area

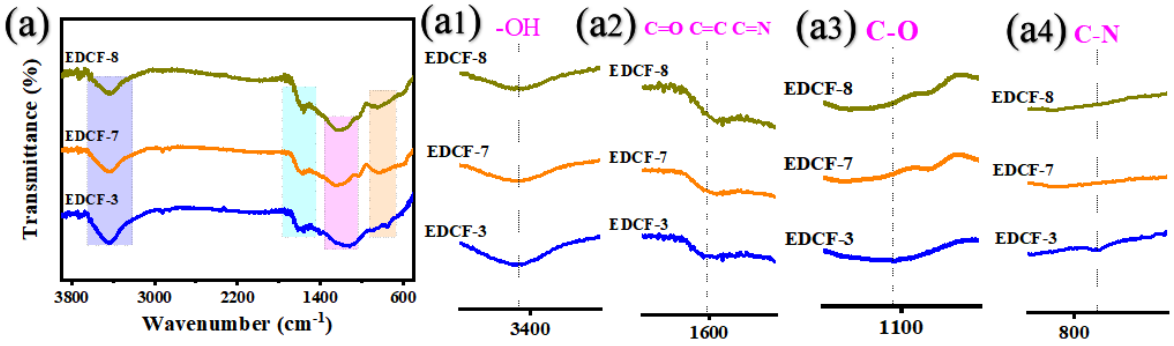

Fig. S16 FT-IR spectra of various EDCF samples with same specific surface area

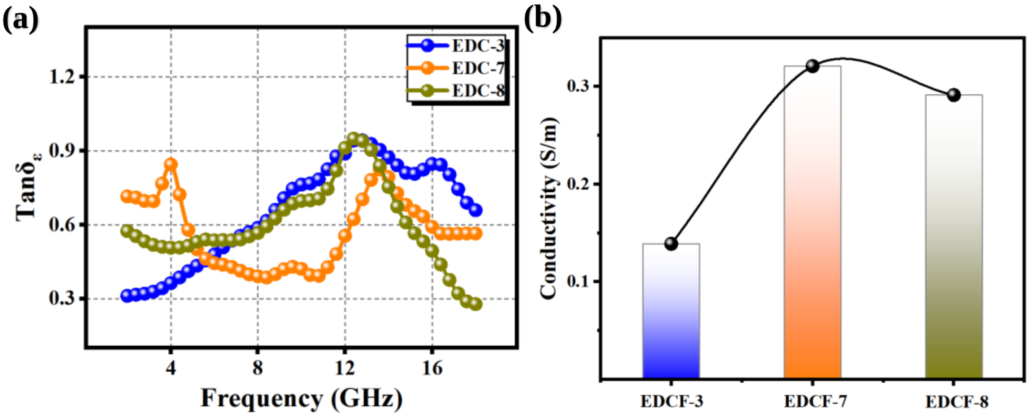

Fig. S17 Tangent of dielectric loss of the EDCF samples(**a**); Conductivity of EDCF-3, EDCF-7 and EDCF-8 (**b**)

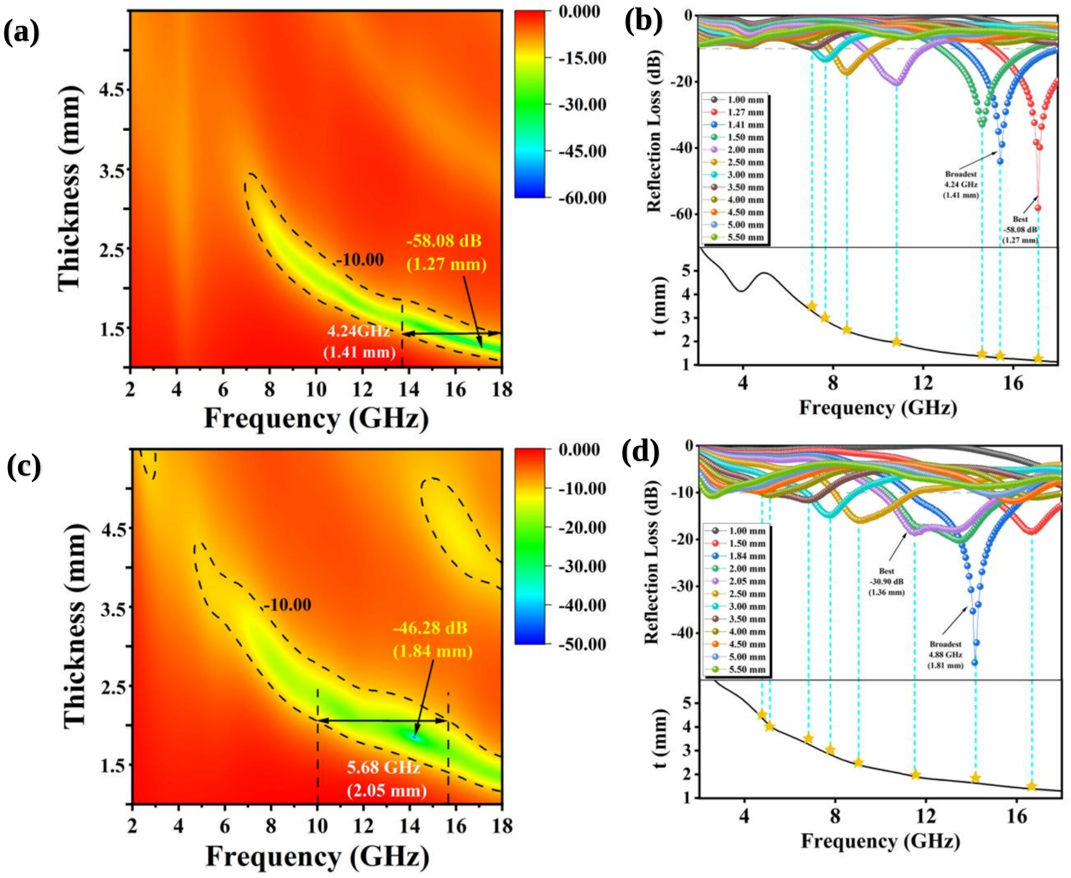

Fig. S18 RL curves of EDCF-7 (**a, b**), EDCF-8 (**c, d**) at different thicknesses of 2~18 GHz

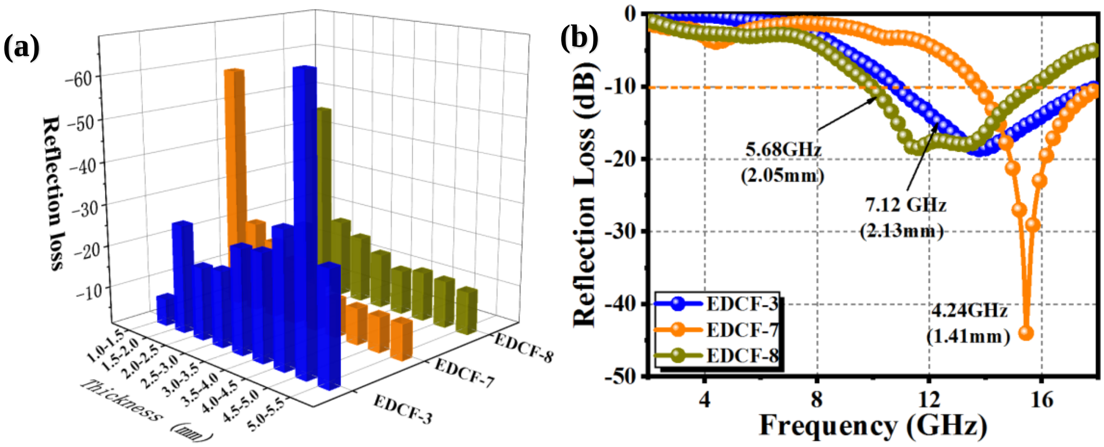

Fig. S19 Comparison of minimum RL value of the samples at different thickness (**a**); Comparison of EAB values of EDCF-3, EDCF-7 and EDCF-8 (**b**)

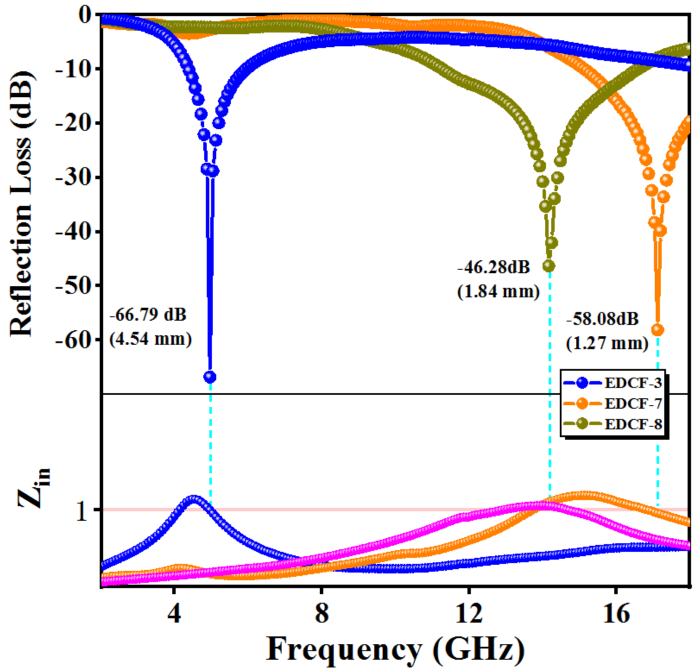

Fig. S20 Normalized impedance matching characteristic of EDCF-3, EDCF-7 and EDCF-8 samples

Table S3 RL_{min} (**a**) and EAB (**b**) versus thickness of carbon-based absorbing materials reported recently

Absorber	Filler content	RL_{min} value		$RL \leq$ -10 dB		Refs.
		d(mm)	RLmin (dB)	d (mm)	EAB(GHz)	
N-doped porous carbon aerogel	20%	2.7	-49.3	2.7	4.5	[S1]
walnut shell-derived nano-porous carbon	30%	2	42.4	1.5	2.24	[S2]
Nanoporous Carbon	9%	1.6	-24	1.9	6	[S3]
MOF-derived PC-based nanocomposites	33%	2	-47.6	2	5.1	[S4]
HPCNs	25%	1.6	-18.13	1.6	5.17	[S5]
GPCN	4%	5.0	-32.7	2.1	6.0	[S6]
porous CZC	30%	3.0	-52.6	3.0	4.9	[S7]
MHPFs	30%	3.4	-55.39	3.4	3.8	[S8]
3D-CFO@CN	20%	2.0	-52.29	2.0	5.36	[S9]

Fe/Fe_3O_4@porous carbon	30%	1.8	-50.05	1.8	5.2	[S10]
MRC-C	15%	1.6	-42.40	1.6	4.37	[S11]
Fe_3O_4@AEWC	20%	2.5	-43.7	2.5	7.52	[S12]
Ag/PC	20%	1.8	-35.4	1.8	2.0	[S13]
PCHN	3%	2.2	-30.46	2.2	5.44	[S14]
SCN	30%	2.2	-54.5	2.2	6.88	[S15]
EDCF-3	5%	4.54	-66.79	2.13	7.12	This work
EDCF-7	5%	1.29	-52.77	1.41	4.24	This work

Supplementary References

[S1] P.B. Liu, S. Gao, C. Chen, F.T. Zhou, Z.Y. Meng et al., Vacancies-engineered and heteroatoms-regulated N-doped porous carbon aerogel for ultrahigh microwave absorption. Carbon **169**, 276-287 (2020). https://doi.org/10.1016/j.carbon.2020.07.063

[S2] X. Qiu, L.X. Wang, H.L. Zhu, Y.K. Guan, Q.T. Zhang, Lightweight and efficient microwave absorbing materials based on walnut shell-derived nano-porous carbon. Nanoscale **9**(22), 7408-7418 (2017). https://doi.org/10.1039/C7NR02628E

[S3] H.Q. Zhao, Y. Cheng, H.L. Lv, B.S. Zhang, G.B. Ji et al., Achieving sustainable ultralight electromagnetic absorber from flour by turning surface morphology of nanoporous carbon. ACS Sustain. Chem. Eng. **6**(11), 15850-15857 (2018). https://doi.org/10.1021/acssuschemeng.8b04461

[S4] J. Li, P. Miao, K.J. Chen, J. Cao, Y. Tang et al., Highly effective electromagnetic wave absorbing prismatic Co/C nanocomposites derived from cubic metal-organic framework. Compos. Part B Eng. **182**, 107613 (2020). https://doi.org/10.1016/j.carbon.2020.10.062

[S5] J.Q. Tao, J.T. Zhou, Z.J. Yao, Z.B. Jiao, B. Wei et al., Multi-shell hollow porous carbon nanoparticles with excellent microwave absorption properties. Carbon **172**, 542-555 (2021). https://doi.org/10.1016/j.carbon.2020.10.062

[S6] H.Q. Zhao, Y. Cheng, Z. Zhang, B.S. Zhang, C.C. Pei et al., Biomass-derived graphene-like porous carbon nanosheets towards ultralight microwave absorption and excellent thermal infrared properties. Carbon **173**, 501-511 (2021). https://doi.org/10.1016/j.carbon.2020.11.035

[S7] Q. Liao, M. He, Y.M. Zhou, S.X. Nie, Y.J. Wang et al., Highly cuboid-shaped heterobimetallic metal-organic frameworks derived from porous Co/ZnO/C microrods with improved electromagnetic wave absorption capabilities. ACS Appl. Mater. **10**(34), 29136-29144 (2018). https://doi.org/10.1021/acsami.8b09093

[S8] F. Wu, Z.L. Liu, T. Xiu, B.L. Zhu, I. Khan, Fabrication of ultralight helical porous carbon fibers with CNTs-confined Ni nanoparticles for enhanced microwave absorption. Compos. Part B Eng. **215**, 108814 (2021). https://doi.org/10.1016/j.compositesb.2021.108814

[S9] R.X. Xu, D.W. Xu, Z. Zeng, D. Liu. $CoFe_2O_4$/porous carbon nanosheet composites for broadband microwave absorption. Chem. Eng. J. **427**, 130796 (2022). https://doi.org/10.1016/j.cej.2021.130796

[S10] X.J. Zhu, Y.Y. Dong, F. Pan, Z. Xiang, Z.C. Liu et al., Covalent organic framework-derived hollow core-shell Fe/Fe_3O_4@porous carbon composites with corrosion

resistance for lightweight and efficient microwave absorption. Compos. Commun. **25**, 100731 (2021). https://doi.org/10.1016/j.coco.2021.100731

[S11] W.T. Yang, X.S. Yang, J.K. Hu, D.Y. Liu, Y.B. Zhu et al., Mushroom cap-shaped porous carbon particles with excellent microwave absorption properties. Appl. Surf. Sci. **564**, 150437 (2021). https://doi.org/10.1016/j.apsusc.2021.150437

[S12] Z.Z. Guo, P.G. Ren, F.D. Zhang, H.J. Duan, Z.Y. Chen et al., Magnetic coupling N self-doped porous carbon derived from biomass with broad absorption bandwidth and high-efficiency microwave absorption. J. Colloid Interf. Sci. **610**, 1077-1087 (2022). https://doi.org/10.1016/j.jcis.2021.11.165

[S13] Y. Lin, C. Ji, L.L. Lu, J. Xu, X.L. Su, Facile synthesis and electromagnetic wave absorption properties of silver coated porous carbon composite materials. J. Alloys Compd. **856**, 158194 (2021). https://doi.org/10.1016/j.jallcom.2020.158194

[S14] S. Wei, Z.C. Shi, W.R. Wei, H.L. Wang, D. Dastan et al., Facile preparation of ultralight porous carbon hollow nanoboxes for electromagnetic wave absorption. Ceram. Int. 47(19), 28014-28020 (2021). https://doi.org/10.1016/j.ceramint.2021.06.132

[S15] B. Wen, H.B. Yang, Y. Lin, L. Ma, Y. Qiu et al., Controlling the heterogeneous interfaces of S, Co co-doped porous carbon nanosheets for enhancing the electromagnetic wave absorption. J. Colloid Interf. Sci. **586**, 208-218 (2021). https://doi.org/10.1016/j.jcis.2020.10.085
